# Maximal Rashba Splitting in GeTe/Bi_2_Te_3_ Heterostructures via Strong Band Bending

**DOI:** 10.1002/advs.202513673

**Published:** 2025-11-07

**Authors:** Qing‐Lin Yang, Xu Yang, Jia‐Wan Li, Yan Li, Jin Tang, Hai‐Feng Du, Zi‐Zhao Gong, Hao‐Pu Xue, Jia‐Nan Liu, Zhuo Deng, Peng‐Tao Yang, Xiang‐Qun Zhang, Wei He, Yusheng Hou, Zhao‐Hua Cheng

**Affiliations:** ^1^ Beijing National Laboratory for Condensed Matter Physics, Institute of Physics Chinese Academy of Sciences Beijing 100190 China; ^2^ Guangdong Provincial Key Laboratory of Magnetoelectric Physics and Devices, Center for Neutron Science and Technology, School of Physics Sun Yat‐Sen University Guangzhou 510275 China; ^3^ School of Physical Sciences University of Chinese Academy of Sciences Beijing 100049 China; ^4^ Anhui Province Key Laboratory of Condensed Matter Physics at Extreme Conditions High Magnetic Field Laboratory of the Chinese Academy of Sciences and University of Science and Technology of China Hefei 230031 China; ^5^ Institute of Physical Science and Information Technology Anhui University Hefei 230601 China; ^6^ Songshan Lake Materials Laboratory Dongguan Guangdong 523808 China

**Keywords:** band bending, band structure, ferroelectric Rashba semiconductors, heterostructure, Rashba effect

## Abstract

The Rashba effect has emerged as a pivotal phenomenon driving novel discoveries in condensed matter physics. Materials with large Rashba energy *E*_R_, wavenumber offset *k*_0_ and Rashba parameter α_R_ are prerequisites for spintronic devices operating above room temperature. While neither ultrathin GeTe films (<3.0 nm thickness) nor monolayer topological insulator Bi_2_Te_3_ (1 quintuple layer (QL) on Si(111)) manifest Rashba splitting, An unprecedented strength of Rashba effect with $E_{R}=\left(0.57\pm 0.03\right)\;\mathrm{eV}$, $k_{0}=\left(0.16\pm 0.03\right){Å}^{-1}$ and $\alpha_{R}=\left(6.86\pm 0.59\right)\mathrm{eV}\cdot Å$ in GeTe (1 nm)/Bi_2_Te_3_ (1 QL) heterostructure is achieved. The spin‐momentum‐locked bands resulting from the Rashba effect of GeTe /Bi_2_Te_3_ is confirmed by density functional theory (DFT) calculations. In GeTe (*x* nm)/Bi_2_Te_3_(10 QL), it is find that the values of *E*_R_, *k*_0_ and α_R_ significantly enhance as the thickness of GeTe decreases contrasting with GeTe films. By comparing the thickness dependence of GeTe with that of GeTe/Bi_2_Te_3_, it is determined that the enhanced Rashba parameter Δα_*R*_ is proportional to the electric field in the GeTe/Bi_2_Te_3_ heterojunction region. It is concluded that the origin of this huge Rashba effect is attributed to the strong band bending in the GeTe/Bi_2_Te_3_ heterojunction region, where the striking inversion‐symmetry‐breaking and significant bandgap difference result in a sharp potential gradient normal to the interface. This work opens an avenue to enhance the Rashba splitting by strong band bending and design spin field effect transistors with spin channel as short as several nanometer scales.

## Graphical abstract

An unprecedented strength of Rashba effect with *E*_R_ = 0.57 eV, $\;k_{0}=0.16\;{Å}^{-1}$ and $\alpha_{R}=6.86\;\mathrm{eV}\cdot Å$ in GeTe (1 nm)/Bi_2_Te_3_ (1 QL) heterostructure. The enhanced Rashba is origined from the band bending effect in the heterojunction region of GeTe /Bi_2_Te_3_ heterostructure, which caused by charge transfer.

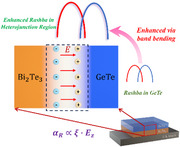

## 1 Introduction

The Rashba spin‐orbit coupling (SOC) induces spin‐splitting bands with spin‐momentum locking in inversion symmetry breaking (ISB) materials, enabling the generation, detection, and manipulation of spin current without external magnetic field.^[^ ^1^, ^2^, ^3^ ^]^ The Rashba effect leads to intriguing physical manifestations and discoveries;^[^ ^4^, ^5^ ^]^ it explores various spintronic devices,^[^ ^6^ ^]^ quantum transport,^[^ ^7^, ^8^ ^]^ and quantum computations with Majorana fermions.^[^ ^7^, ^9^ ^]^ Furthermore, it paves a new path to enhance thermoelectric performance.^[^ ^10^ ^]^ Materials with a strong Rashba effect, quantified by three parameters $E_{R}>26\;\mathrm{meV},\;k_{0}>0.1\;{Å}^{-1},\alpha_{R}>3$ eV·Å are highly desirable for designing various spintronic devices in favor of room‐temperature operations, including spin field effect transistors with shorter spin channel length, highly efficient spin‐charge converters, as well as non‐reciprocal rectification devices.^[^ ^4^, ^11^, ^12^, ^13^, ^14^, ^15^, ^16^ ^]^

The Rashba splitting is determined by the interplay between SOC and ISB,^[^ ^17^ ^]^ has been extensively investigated across diverse material systems, including two‐dimensional electron gas,^[^ ^1^, ^18^, ^19^, ^20^, ^21^ ^]^ organic‐inorganic perovskites,^[^ ^22^, ^23^, ^24^, ^25^, ^26^ ^]^ topological insulators, superconductors,^[^ ^18^, ^27^, ^28^ ^]^ and the ferroelectric Rashba semiconductors (FERSCs).^[^ ^29^, ^30^, ^31^, ^32^ ^]^ Both theoretical and experimental works prove that the Rashba parameter α_*R*_ is proportional to the strength of SOC ξ and the electric field *E_Z_* normal to the surface.^[^ ^1^, ^4^, ^33^, ^34^, ^35^, ^36^ ^]^ Therefore, the Rashba splitting size can be manipulated by an external gate voltage,^[^ ^3^, ^13^, ^37^, ^38^, ^39^ ^]^ the thickness of the sample,^[^ ^40^ ^]^ element doping,^[^ ^41^ ^]^ and an ultrafast laser pulse.^[^ ^42^ ^]^ For a heterostructure system, fabricating the ISB interface with a strong SOC semiconducting material represents an effective strategy for simultaneously enhancing Rashba effects and modifying the band structure.^[^ ^35^, ^43^, ^44^, ^45^, ^46^ ^]^ The ISB, which relates to the electric potential variation in the junction region, can originate from the band bending induced by the substrate, or alternatively from different environments of the two surfaces.^[^ ^47^ ^]^

Recently, GeTe, as a prototypical FERSC, has garnered widespread attention due to its large Rashba parameter ($\alpha_{R}\approx \;4.8\;\mathrm{eV}\;Å$).^[^ ^30^ ^]^ However, this distinct Rashba band structure disappears when the thickness falls below 3.0 nm. This so‐called three‐dimensional limit of bulk Rashba effect in GeTe hinders the application of device requiring extreme miniaturization.^[^ ^40^ ^]^ Consequently, exploring a new method to create a large Rashba splitting in thin GeTe film is a crucial objective.^[^ ^48^ ^]^

Here, we construct a heterointerface composed of two materials with significant difference in bandgaps and opposite carrier types: GeTe and Bi_2_Te_3_. Although angle‐resolved photoemission spectroscopy (ARPES) spectra demonstrate that GeTe thin films with a thickness of less than 3.0 nm and Bi_2_Te_3_ with a thickness of 1 quintuple layer (QL, 1QL≈ 1.02 nm)/Si (111) do not exhibit Rashba splitting, we achieve an unprecedented strength of Rashba effect with *E*_R_ = (0.57 ± 0.03) eV, $\;k_{0}=\left(0.16\pm 0.03\right){Å}^{-1}$ and $\alpha_{R}=\left(6.86\pm 0.59\right)\;\mathrm{eV}\cdot Å$ in GeTe (1 nm)/Bi_2_Te_3_(1 QL) heterostructure. Moreover, the band structure of GeTe /Bi_2_Te_3_ heterostructure, calculated using density functional theory (DFT), reveals a giant Rashba splitting of α_R_ = 6.71 eV·Å, and the band exhibits a spin‐momentum locking feature. This giant Rashba effect originates from strong band bending owing to the significant difference in bandgaps of 720 meV and 165 meV of the *p*‐type GeTe and *n*‐type Bi_2_Te_3_. Our findings offer a novel means to enhance the Rashba effect though strong band bending and to design shorter spin channel devices capable of operating above room‐temperature.

## 2 Results and Discussion

**Figure 1:**
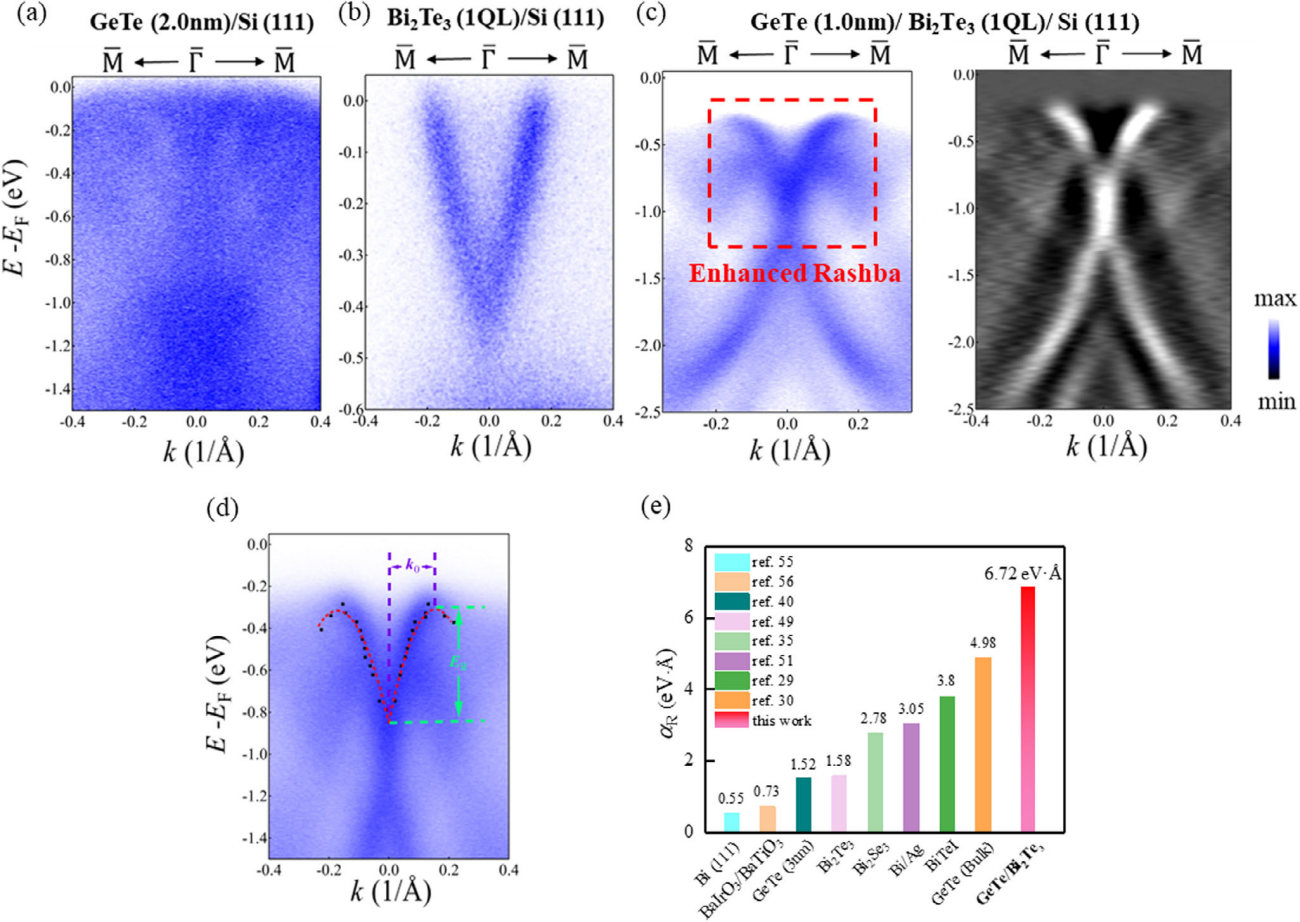
**a)** ARPES spectrum of GeTe (2.0 nm)/Si (111). **b)** ARPES spectrum of Bi_2_Te_3_ (1 QL)/Si (111). **c)** ARPES spectrum of GeTe (1.0 nm)/Bi_2_Te_3_ (1 QL) /Si (111) heterostructure and its second derivative. d) The fitting result of the ARPES spectrum of GeTe (1.0 nm)/Bi_2_Te_3_ (1 QL) /Si (111) heterostructure to obtain α_R_. The black dots are the peak positions of the momentum distribution curves (MDCs). The red dashed line is the fitting lines of the Rashba band structures. e) Rashba parameters α_*R*_ for representative Rashba materials.^[^ ^29^, ^30^, ^35^, ^40^, ^49^, ^51^, ^55^, ^56^ ^]^

**Figure** **1**a shows the ARPES result of 2.0 nm GeTe, where no detectable Rashba splitting bands are observed. The significant lattice mismatch between Si and GeTe broadens the ARPES spectrum of GeTe (2.0 nm)/Si. This finding is in good agreement with the three‐dimensional limit, i.e., the bulk Rashba effect will disappear when the thickness of GeTe decreases below 3.0 nm.^[^ ^40^ ^]^ Croes et al. reported a reinforced Rashba effect in ultrathin GeTe via 5–10% Sb doping. Sharper spectra was reported for GeTe grown on Sb‐terminated Si (111). Sb doping likely stabilizes the non‐centrosymmetric lattice structure of GeTe, facilitating the *preservation* of its inherent Rashba effect.^[^ ^48^ ^]^ Figure 1b displays the ARPES result of 1 QL Bi_2_Te_3_, which also lacks distinct Rashba band structures. Although the Rashba coupling parameter of bulk Bi_2_Te_3_ (156 nm thickness) epitaxial film was reported to be α_R_ = 1.58 eV·Å via Shubnikov–de Haas oscillations measurement, the corresponding Rashba splitting in energy and momentum space are only *E*_R_ = 6.8 meV and *k*_0_ = 0.009 Å, respectively, which are too small to be resolved by conventional ARPES.^[^ ^49^ ^]^ Given the opposite carrier types of GeTe and Bi_2_Te_3_, as confirmed by the ARPES spectra of bare GeTe and Bi_2_Te_3_ in supplemental information, we fabricated the samples of GeTe (*x* nm)/Bi_2_Te_3_ (*y* QL) heterostructures via molecular beam epitaxy (MBE) on Si (111) substrates. Details regarding sample quality can be found in supplemental information (Figure S1, Supporting Information). To explore the potential application of GeTe (*x* nm)/Bi_2_Te_3_ (*y* QL) heterostructures in low dimensional devices, it is crucial to minimize the thickness of both GeTe, *x* and Bi_2_Te_3_, *y*, simultaneously. Since a 1 QL Bi_2_Te_3_ interlayer similarly mitigates lattice mismatch, our work demonstrates substantially sharper ARPES spectra in GeTe/Bi_2_Te_3_/Si (111) heterostructures. Figure 1c demonstrates that GeTe (1 nm)/Bi_2_Te_3_ (1 QL) heterostructure exhibits a pronounced Rashba splitting band in the direction of $\bar{M}-\bar{\Gamma }-\bar{M}$, exceeding the so‐called 3D limit of GeTe bulk Rashba effect via epitaxial growth onto Bi_2_Te_3_. Although spectral broadening persists in the ultrathin limit—increasing experimental uncertainty—the ARPES data for GeTe (1 nm)/Bi_2_Te_3_ (1 QL) and its second derivative reveal a resolvable Rashba‐split band. This clarity allows unambiguous extraction of Rashba parameters.

The Hamiltonian of a system with Rashba effect can be described by $H=\frac{\hbar^{2}k^{2}}{2m^{*}}+\alpha_{R}\left(\hat{z}\times k\right)\cdot \sigma .$ ^[^ ^50^ ^]^ As a result, the Rashba split band could be fitted by the dispersion relation:^[^ ^50^ ^]^

(1) $$E_{\pm }=\frac{\hbar^{2}k^{2}}{2m^{*}}\pm \alpha_{R}\left|k\right|+E_{0}$$

where ℏ is the reduced Planck constant. m* is the effective mass of the carriers. α_R_ is the Rashba parameter, which contains all material‐dependent parameters (in this case the surface potential gradient), and *E*_0_ is the energy of the band edge.

We cut the momentum distribution curves (MDCs) of the ARPES spectra and extract the peak position by Lorentzian function fitting. The MDCs and the corresponding fitting results are displayed in Figure S2 (Supporting Information). As illustrated in Figure 1d, the black dots represent the extracted peak positions and the red dashed line indicates the fitting line of the Rashba band structure using Equation (1). The fitted value of the Rashba coupling parameter is $\alpha_{R}=\left(6.86\pm 0.59\right)\;\mathrm{eV}\cdot Å$ along the direction of $\bar{M}-\bar{\Gamma }-\bar{M}$. Besides, the effective mass m* = (− 0.18 ± 0.02)*m_e_*, *m_e_* is the mass of a free electron, *E*_0_ = (− 0.88 ± 0.04)eV. The reduced chi‐square is 0.001 and the R‐square is 0.966 for this fitting, indicating a good fit. The Rashba effect can also be quantified by the Rashba energy of split states *E*_R_ and the wave number offset *k*_0_, as shown in Figure 1d.^[^ ^51^ ^]^ The obtained values are *E*_R_ = (0.57 ± 0.03) eV and $\;k_{0}=\left(0.16\pm 0.03\right){Å}^{-1}$. Both values surpass those of other Rashba systems.^[^ ^29^ ^]^ The large Rashba energy *E*_R_ ≫ *k*_B_ *T* (≈ 26 meV for room temperature) is critical for operating spintronic devices above room temperature. More interestingly, the large wavenumber offset $\left(k_{0}=0.16\;{Å}^{-1}\right)$ is a key parameter for reducing the spin channel length of spin field effect transistors down to several nanometer scales (*L* = π/2*k*_0_ ≈ 1 nm).^[^ ^15^, ^52^ ^]^ Furthermore, we measured the ARPES spectrum along the cross direction $\bar{K}-\bar{\Gamma }-\bar{K}$, which is displayed in Figure S10a (Supporting Information). The significant Rashba splitting persists, with the Rashba coupling parameter $\alpha_{R}=\left(6.80\pm 1.13\right)\mathrm{eV}\cdot Å\;$ along $\bar{K}-\bar{\Gamma }-\bar{K}$, which is close to the value along the direction of $\bar{M}-\bar{\Gamma }-\bar{M}$. By comparing with Rashba parameters α_*R*_ for representative Rashba materials, such as Bi(111), Bi/Ag, FERSCs, bulk GeTe, BiTeI, and oxide interfaces,^[^ ^29^, ^30^, ^35^, ^40^, ^49^, ^51^, ^53^, ^54^, ^55^, ^56^ ^]^ we find that GeTe/Bi_2_Te_3_ possesses an unprecedented magnitude of Rashba coupling parameter α_R_(Figure 1e). The transport measurements also reveal the presence of stronger Rashba spin‐orbit coupling when the heterostructure thickness is low. In addition to the large α_R_, the Fermi level is located at the band gap in Figure 1d, indicating the heterostructure's semiconducting nature, which is also confirmed by transport measurements. Further details are provided in the supplementary information (Figures S3,S4, Supporting Information).

**Figure 2:**
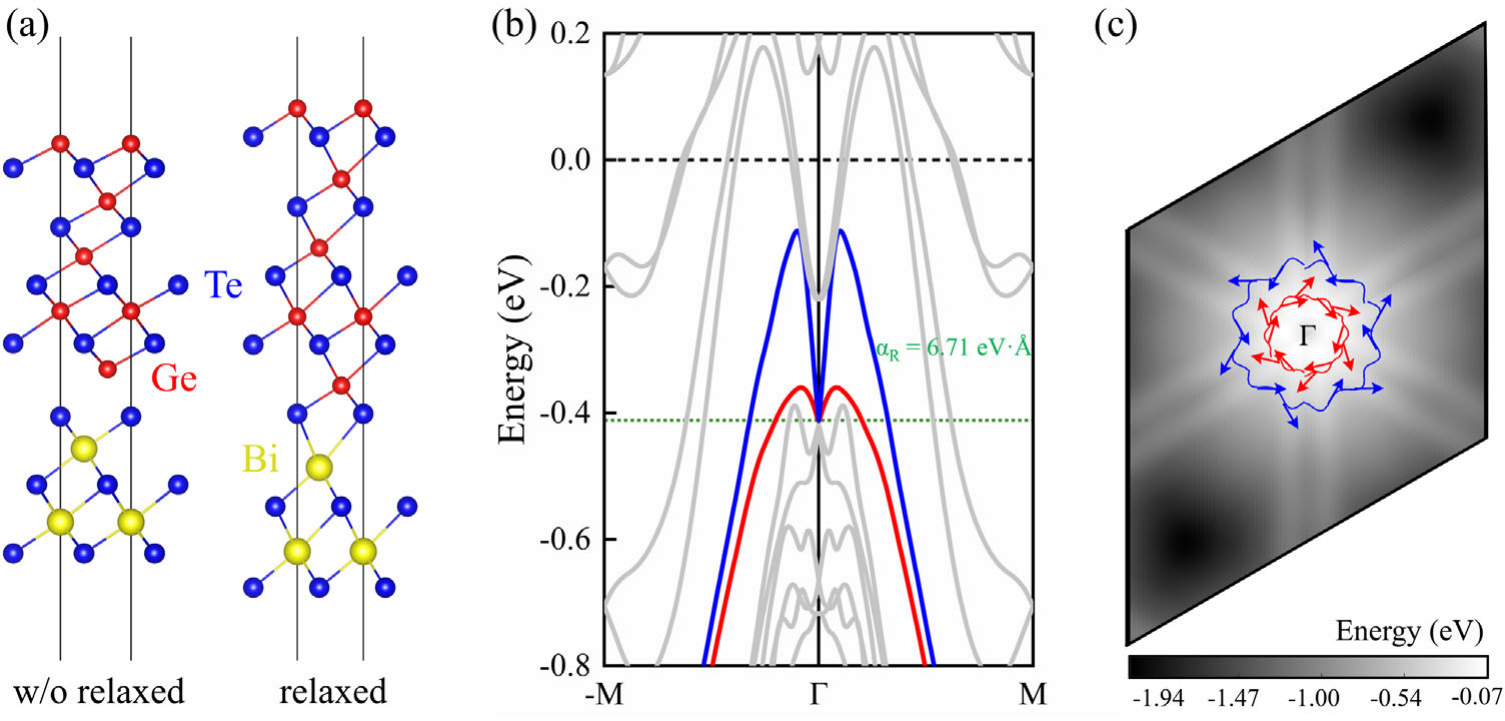
**a)** Atomic structure of the GeTe/Bi_2_Te_3_ (1QL) heterostructure with the *fcc* configuration. The left (right) panel shows the unrelaxed initial (relaxed) structure. **b)** DFT calculated band structure along the $\bar{M}-\bar{\Gamma }-\bar{M}$ high‐symmetry path. Rashba‐split bands are indicated by the red and blue curves, and their crossing point at ‐0.412 eV below the Fermi level are indicated by the green dotted line. **c)** 2D projection of Rashba bands. The color scale represents energy distribution. In‐plane spin texture at ‐0.412 eV below the Fermi level is shown by constant energy contours, with arrows indicating spin orientation and magnitude.

To gain a deeper insight into the experimentally observed Rashba splitting, we construct a GeTe/Bi_2_Te_3_(1QL) heterostructure with a *fcc* configuration, as shown in **Figure** **2**a, and investigate its electronic properties using DFT calculations. Although the initial interface van der Waals gap between GeTe and 1QL‐ Bi_2_Te_3_ is set as 2.56 Å, structural relaxations lead to the Te atoms of 1QL‐ Bi_2_Te_3_ bonding with the Ge atom of GeTe. This suggests strong interfacial interactions between GeTe and 1QL‐Bi_2_Te_3_ in GeTe/Bi_2_Te_3_ (1QL). Figure 2b presents the DFT calculated band structure of the relaxed GeTe/Bi_2_Te_3_ (1QL) heterostructure. It indicates a metallic character with multiple bands crossing the Fermi level, which differs from the semiconductor behavior in our ARPES observed bands. This discrepancy may stem from the possible interfacial reconstruction in our experimental samples, which is not accounted for in our DFT model. Nevertheless, the calculated band structure reveals Rashba‐split bands located at 0.412 eV below the Fermi level, as shown in Figure 2c, with clear spin‐momentum locking. Additional DFT calculations with and without SOC of either GeTe or Bi_2_Te_3_ (see Figure S6, Supporting Information) further confirm that the Rashba splitting vanishes when the SOC is switched off, demonstrating that the inclusion of SOC, especially the SOC of GeTe, is essential for reproducing the observed splitting. Notably, the calculated Rashba parameter of 6.71 eV·Å, is in good agreement with the experimentally observed value. Our further DFT calculations demonstrate that interfacial reconstructions which may occur in experimental samples open a band gap along the $\bar{M}-\bar{\Gamma }-\bar{M}$ path, suggesting a tendency toward insulating behavior, and qualitatively maintain the Rashba‐type spin splitting and spin‐momentum locking, although the Rashba parameter is weakly reduced (as discussed in Figure S5, Supporting Information).

**Figure 3:**
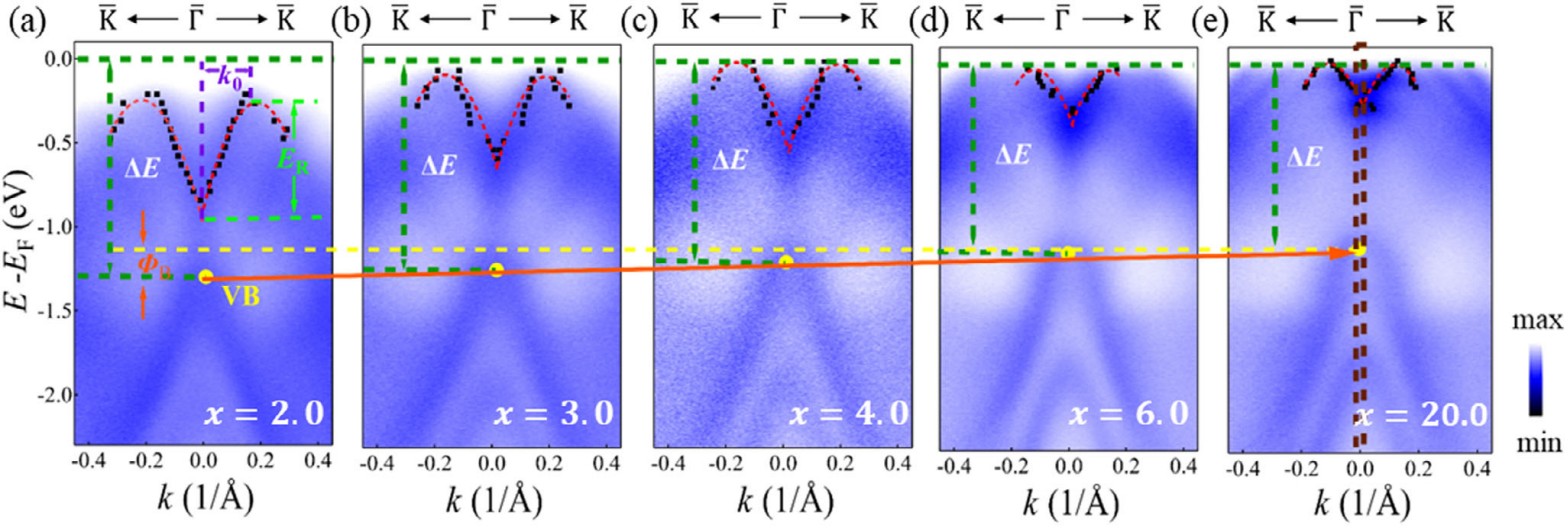
**a–e)** ARPES spectra of GeTe (*x* nm)/Bi_2_Te_3_ (10 QL) heterostructures with the thickness of GeTe, *x* = 2.0, 3.0, 4.0, 6.0, and 20.0 nm, respectively. The black dots represent the peak positions of the momentum distribution curves (MDCs). The red dashed lines are the fitted lines for Rashba bands. The Rashba energy *E*_R_, wavenumber offset *k*_0_, interfacial electric potential ϕ _b_ are represented by the purple, green and orange arrows, respectively.

**Figure 4:**
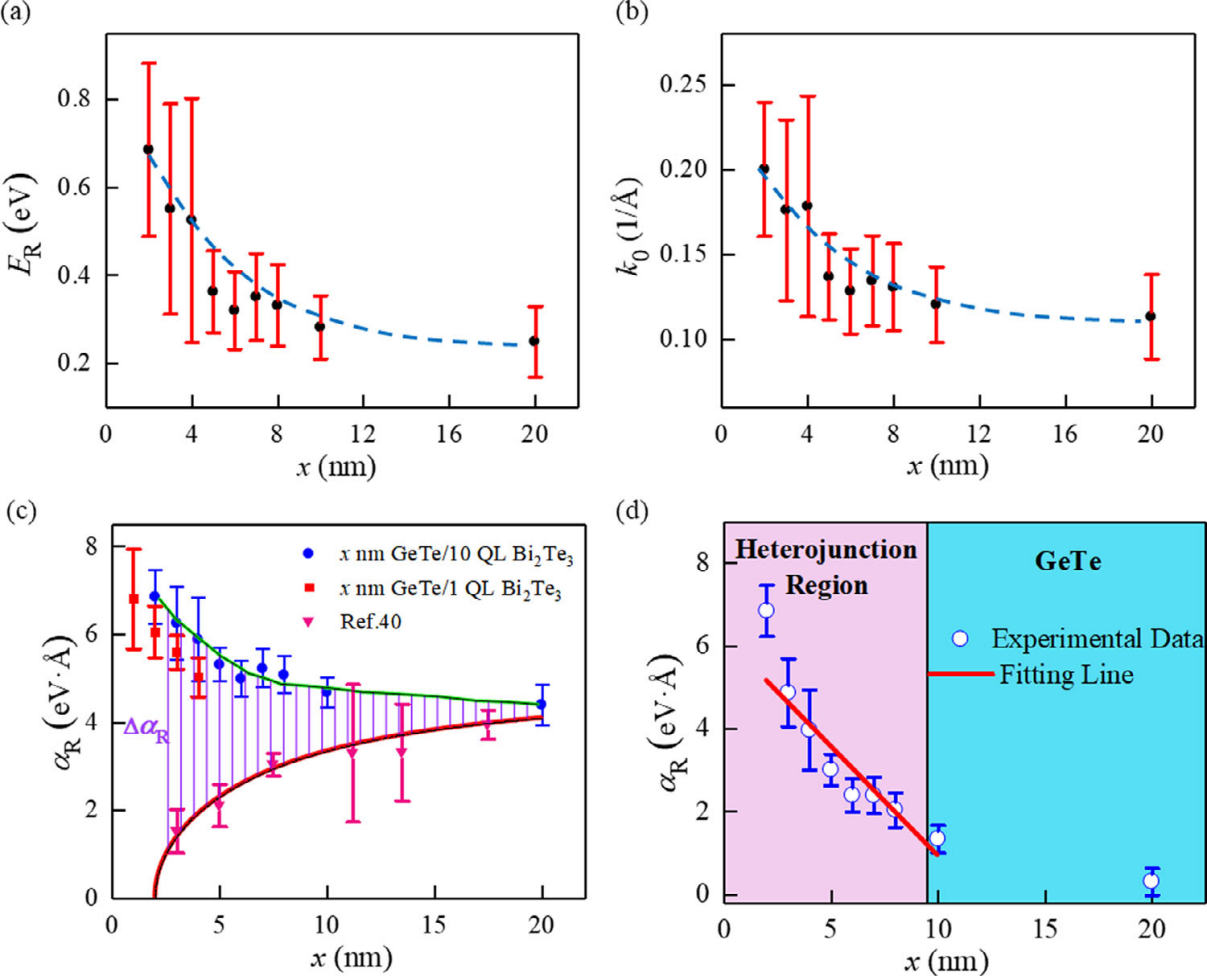
GeTe thickness dependence of Rashba effect of GeTe (*x* nm)/Bi_2_Te_3_ (*y* QL) heterostructures, *y* = 1 and 10). **a)** *E*_R_, **b)** *k*_0_, **c)** Rashba parameter α_R_, **d)** Δα_R_ as a function of *x*. For comparison, the Rashba parameters of bare GeTe (*x* nm)/Si (111) are also listed in (c).^[^ ^40^ ^]^

We performed ARPES measurements on GeTe (*x* nm)/Bi_2_Te_3_ (*y* QL) heterostructures with varying GeTe thicknesses. The thickness of Bi_2_Te_3_ was set to *y* = 10 QL or 1 QL for comparison. **Figure** **3**a–e depict the ARPES spectra of GeTe (*x* nm)/Bi_2_Te_3_ (10 QL) heterostructures with *x* = 2.0, 3.0, 4.0, 6.0 and 20.0 nm, respectively. The second derivative of the ARPES spectra in Figure 3 are displayed in Figure S8 (Supporting Information) to show the evolutionary trend. Spectra for samples with other GeTe thicknesses are displayed in Figure S9 (Supporting Information). The peak positions were extracted by MDCs curves, and the parameter Rashba energy of split states *E*_R_, the wave number offset *k*_0_, Rashba parameters α_R_ were obtained as discussed previously. The results for *E*_R_ and *k*_0_ are shown in **Figure** **4**a,b. Both parameters increase significantly with decreasing GeTe thickness. In addition, the results of α_*R*_ are illustrated with the blue dots in Figure 4c. For the GeTe (*x* nm)/Bi_2_Te_3_ (*y* QL) heterostructure with *x* = 2.0 nm, the value of α_R_ = 6.26eV·Å, is still far larger than that of bulk GeTe. As the GeTe thickness increases up to *x* = 5.0 nm, α_R_ decreases gradually to 5.31 eV·Å, close to that of bulk GeTe, and remains nearly constant with further increasing *x*. Figures S10a—d (Supporting Information) display the ARPES spectra of GeTe (*x* nm)/Bi_2_Te_3_ (1 QL) heterostructures with different GeTe thicknesses of *x* = 1.0, 2.0, 3.0, and 4.0 nm along the direction of $\bar{K}-\bar{\Gamma }-\bar{K}$. A similar enhancement of the Rashba effect is also observed with decreasing *x*. For comparison, the thickness dependence of Rashba coupling parameters of bare GeTe (*x* nm)/Si (111) is also plotted in Figure 4c.^[^ ^40^ ^]^ The Rashba coupling parameter decreases with the thickness. A striking difference in the thickness‐dependent Rashba effect between GeTe (*x* nm)/Bi_2_Te_3_ (*y* QL) heterostructures and bare GeTe (*x* nm)/Si (111) is evident. By comparing the thickness dependence of GeTe with that of GeTe/Bi_2_Te_3_, the enhancement of the Rashba parameter Δα_R_ = α_Rhet_ − α_RGeTe_ increases linearly with decreasing *x* when *x*< 10 nm (Figure 4d).

The unexpected enhancement of the Rashba effect in GeTe (*x* nm)/Bi_2_Te_3_ (*y* QL) heterostructures is attributed to the SOC and ISB.^[^ ^1^ ^]^ The breaking of structural inversion symmetry can stem from the band bending or different environments of the two surfaces. When the film is much thinner than the thickness of the band‐bending region, the potential variation along the *z‐*direction of the film will become smoother. Consequently, the Rashba effect, as observed in 2D electron gas and semiconductor heterojunctions,^[^ ^33^, ^34^ ^]^ is generally weaker in thinner films. However, in the case of GeTe/Bi_2_Te_3_ heterostructures, the significant difference in bandgaps between GeTe and Bi_2_Te_3_ suggests that a giant Rashba splitting can be achieved due to the sharp potential gradient along the normal to the interface via strong band bending. Due to the opposite carrier types of GeTe and Bi_2_Te_3_ as shown in Figure S7 (Supporting Information), the charge transfer at the interface of GeTe (*x* nm)/Bi_2_Te_3_ (*y* QL) heterostructures can cause the Fermi level to shift relative to the conduction band. For bare GeTe, its Fermi level lies within the valence band (Figure S7a (Supporting Information), whereas the ARPES spectrum demonstrates the Fermi level of GeTe (1.0 nm)/Bi_2_Te_3_ (1 QL) is situated in the bandgap (Figure 1c). This upward shift in Fermi energy indicates charge transfer from Bi_2_Te_3_ to GeTe. Since thinner Bi_2_Te_3_ can only provide less charge, the Fermi levels of GeTe (*x* nm)/Bi_2_Te_3_ (1 QL) heterostructures are higher and the Rashba parameters α_R_ are smaller than those of GeTe (*x* nm)/Bi_2_Te_3_ (10 QL) heterostructures with same thickness of GeTe, *x* (Figure 4c).

To confirm the position of the valence band (VB), integrated energy distribution curves (EDCs) near $\bar{\Gamma }$ (as shown in Figure 3e) for different GeTe thicknesses were extracted and exhibited in Figure S10a (Supporting Information). The position of VB could be confirmed by multiple peaks fitting with the Lorentzian function, as represented by the black tick marks in Figure S10a (Supporting Information). The Fermi level was characterized by referencing the position of the VB at deeper energies, represented by yellow dots in Figure 3a–e. With increasing the thickness of GeTe, the position of VB shifts upward, indicating that the Fermi level moves downwards. The relative position of the Fermi level, expressed by Δ*E*_V_ = *E*_VB_ − *E*_F_, moves downward with increasing GeTe thickness, *x*, signifying the charge transfer from Bi_2_Te_3_ to GeTe and a band bending at the interface of GeTe (*x* nm)/Bi_2_Te_3_ (*y* QL) heterostructures. While the semiconductor p‐n junction model applies to layers thicker than their depletion regions, ARPES's surface sensitivity poses a critical limitation: for such systems, the Fermi level position near the interface becomes experimentally inaccessible. To resolve this, we employ a well‐established thickness‐dependent methodology—varying GeTe film thickness while measuring Fermi level evolution via ARPES. This approach, validated by prior studies of interfacial band alignment in low‐dimensional systems.^[^ ^57^, ^58^, ^59^, ^60^ ^]^

**Figure 5:**
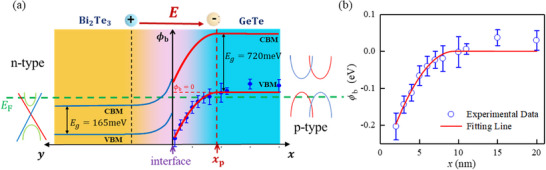
**a)** Schematic illustration of band bending of GeTe/ Bi_2_Te_3_ heterostructure. **b)** Electric potential ϕ_b_ of GeTe (*x* nm)/Bi_2_Te_3_ (10 QL) heterostructures as a function of GeTe thickness, *x*.

The schematic of energy band bending is shown in **Figure** **5**a. In the heterojunction region, the interfacial electric potential ϕ_b_ (as shown in Figure 5a) is related to the distance between the interface, and it could be described by the following equation.^[^ ^58^ ^]^

(2) $$\phi_{b}\left(x\right)=\left\{\begin{array}{cc} -\frac{q^{2}N_{D}}{2\varepsilon_{r}\varepsilon_{0}}{\left(x-x_{p}\right)}^{2} & 0\leq x\leq x_{p}\\ 0 & x>x_{p} \end{array}\right.$$

where *q* is the electron charge, *x*_p_ is the heterojunction width, *N*_D_ is the donor concentration, ε_*r*_ is the relative dielectric constant, ε_0_ is the vacuum dielectric constant, and ϕ_b_(0) is the interfacial electric potential at *x* = 0. Since ϕ_b_ represents the relative shift of the VB position in the heterojunction region, the value of ϕ_b_ for different thicknesses can be calculated by using the VB position subtracting that of bulk GeTe, which is displayed in Figure S10b (Supporting Information), represented by the purple star. The thickness dependence of ϕ_*b*_ is exhibited in Figure 5b, and the parameters of *x*_p_ and ϕ_b_(0) were fitted using Equation (2), where $\frac{q^{2}N_{D}}{2\varepsilon_{r}\varepsilon_{0}}$ is considered as a constant. The fitted values are *x*_p_ = 9.5± 0.9 nm and ϕ_*b*_(0) = ‐317 meV.

The charge transfer at the interface generates a built‐in electric field in the heterojunction region,^[^ ^61^ ^]^ which could enhance *E_Z_*. In the case of GeTe (*x* nm)/Bi_2_Te_3_ (*y* QL) heterostructures, the electric field at the interface associated with the Rashba parameter has two components: the intrinsic polarization *E*_GeTe_ and the one caused by charge transfer *E*_charge transfer_. i.e., *E*_tot_ = *E*_GeTe_ + *E*_charge transfer_. Both components will contribute to the α_*R*_ independently, which could be expressed by α_R GeTe_ and α_R charge transfer_, therefore, α_*R* tot_ = α_*R* GeTe_ + α_*R* charge transfer_. If we assume that the strength of SOC ξ is a constant, the relationship α_R tot_ − α_R GeTe_∝*E*_tot_ − *E*_GeTe_ can be derived. By subtracting the α_*R*_ of bare GeTe from the α_*R*_ of heterostructures, the enhanced part caused by charge transfer can be extracted. In Figure 4c, the purple shadow area represents Δα_R_ = α_Rhet_ − α_RGeTe_, the result is displayed in Figure 4d. Because the electric potential ϕ_b_(*x*)∝(*x* − *x*_p_)^2^, the electric field led by charge transfer will have the relation $E\left(x\right)=\frac{d\phi_{b}\left(x\right)}{dx}\propto x-x_{p}$, suggesting that the strength of electric field reduces linearly with the thickness of GeTe. Consequently, the Rashba parameter caused by charge transfer will also linearly decrease. In Figure 4d, the linear relationship between Δα_R_ and *x* can be confirmed when *x* < 10.0 nm (pink area). When the thickness exceeds 10 nm, the charge transfer should make no difference, the Rashba parameter still larger than normal GeTe, this deviation can be attributed to the change in critical thickness resulting from different lattice mismatches. In addition to the enhanced Rashba effect, we also found that the Fermi level of GeTe (1 nm)/Bi_2_Te_3_ (1 QL) heterostructure is located at the gap, indicating the reduction of GeTe conductivity, which makes it easier to modulate by the gate voltage compared to the pure GeTe.^[^ ^13^, ^62^, ^63^ ^]^ Besides the strong SOC and charge transfer, the reduction of conductivity might also benefit the enhanced Rashba effect. Combined with the reported calculation results, potential candidates such as MoS_2_/Bi_2_Te_3_, Sb/Bi_2_Te_3_/Sb, and Sb/Sb_2_Te_3_/Sb heterojunctions are expected to exhibit giant Rashba splitting.^[^ ^43^, ^46^ ^]^

## 3 Conclusion

In summary, we achieve an unprecedented magnitude of Rashba parameter $\alpha_{R}=\left(6.86\pm 0.59\right)\;\mathrm{eV}\cdot Å$ in GeTe (1 nm)/Bi_2_Te_3_(1 QL) heterostructure though strong band bending. A sharp potential variation along the normal to the interface of GeTe/Bi_2_Te_3_ heterostructures owing to the significant difference in bandgap between GeTe (≈ 720 meV) and Bi_2_Te_3_ (≈ 165 meV) results in a huge electric field in the heterojunction region of heterostructure, thereby leading to in a huge Rashba splitting with the spin‐momentum locking feature. Furthermore, the large Rashba energy *E*_R_ = (0.57 ± 0.03) eV and wavenumber offset $k_{0}=\left(0.16\pm 0.03\right){Å}^{-1}$ render GeTe/Bi_2_Te_3_ heterostructures ideal for designing spin field effect transistors with shorter spin channel length  and for operating spintronic devices above room temperature. Our work provides a novel technique to achieve giant Rashba splitting through strong band bending and design two‐dimensional spintronic devices.

## 4 Experimental Section

### Sample Fabrications

GeTe (*x* nm)/Bi_2_Te_3_ (*y* QL) heterostructures were grown by molecular beam epitaxy (MBE) on Si (111) substrates. The base pressure of the growth chamber was approximately 1.0 × 10^−10^ mbar. Initially, the *n*‐type Si (111) substrates were prepared by resistive heating at 1150 °C to obtain a well‐ordered Si (111)‐7×7 surface. Bi_2_Te_3_ thin films were grown by evaporation of high purity Bi (99.999%) and Te (99.999%) from Knudsen cells onto Si (111).^[^ ^64^ ^]^ The evaporating temperature of Bi and Te were 395, 290 °C, respectively. Subsequently, the GeTe was deposited on top of Bi_2_Te_3_. GeTe films were also grown by co‐evaporating Ge (99.99%) and Te. The evaporating temperature of Ge and Te were 1140, 290 °C, respectively. Using ex situ small‐angle x‐ray reflection (XRR), the growth rate for Bi_2_Te_3_ and GeTe were determined to be 0.33 QL/min and 0.67 nm/min, respectively.

### ARPES Measurements

The electronic structures of GeTe/Bi_2_Te_3_ heterostructures were investigated using in situ angle‐resolved photoemission spectroscopy (ARPES) with a photon energy of 21.2 eV at room temperature. The base pressure of the ARPES chamber was approximately 3.0 × 10^−11^mbar. The energy and momentum resolution of the Scienta DA30L electron energy analyzer was better than 20 meV and 0.01 Å^−1^, respectively.

### Computational Details

First‐principles calculations, based on density‐functional theory, were performed using the Vienna ab initio Simulation Package (VASP).^[^ ^65^ ^]^ The exchange‐correlation potential was treated within the generalized gradient approximation (GGA) using the Perdew‐Burke‐Ernzerhof (PBE) functional.^[^ ^66^ ^]^ The valence electron configurations were set as Bi6*s* ^2^6*p* ^3^, Ge4*s* ^2^4*p* ^2^, and Te5*s* ^2^5*p* ^4^. Core‐valence interactions were described by projector augmented‐wave (PAW) pseudopotentials.^[^ ^67^, ^68^ ^]^ A plane‐wave energy cutoff of 350 eV was used. A stacking configurations of GeTe/Bi_2_Te_3_ (1 QL) heterostructures were built, which was a face‐centered cubic (*fcc*) configuration with Ge atoms positioned above Te atoms of Bi_2_Te_3_ in Figure 2. A vacuum space of 15 Å was added along the normal axis to eliminate spurious interactions between adjacent slabs. The structure was fully optimized until the force on each atom was smaller than 0.01 eV/Å. The van der Waals interactions were described using the semiempirical DFT‐D2 method,^[^ ^69^ ^]^ and spin‐orbit coupling (SOC) was included in all band structure calculations.

## Conflict of Interest

The authors declare no conflict of interest.

## Supporting information



Supporting Information

## Data Availability

The data that support the findings of this study are available from the corresponding author upon reasonable request.

## References

[1] Y. A. Bychkov , E. I. Rashba , JETP Lett. 1984, 39, 78.

[2] A. Manchon , H. C. Koo , J. Nitta , S. M. Frolov , R. A. Duine , Nat. Mater. 2015, 14, 871. 26288976 10.1038/nmat4360

[3] A. Soumyanarayanan , N. Reyren , A. Fert , C. Panagopoulos , Nature 2016, 539, 509. 27882972 10.1038/nature19820

[4] G. Bihlmayer , P. Noël , D. V. Vyalikh , E. V. Chulkov , A. Manchon , Nat. Rev. Phys. 2022, 4, 642.

[5] A. El Hamdi , J.‐Y. Chauleau , M. Boselli , C. Thibault , C. Gorini , A. Smogunov , C. Barreteau , S. Gariglio , J.‐M. Triscone , M. Viret , Nat. Phys. 2023, 19, 1855.

[6] H. C. Koo , S. B. Kim , H. Kim , T. E. Park , J. W. Choi , K. W. Kim , G. Go , J. H. Oh , D. K. Lee , E. S. Park , I. S. Hong , K. J. Lee , Adv. Mater. 2020, 32, 2002117. 10.1002/adma.202002117 32930418

[7] D. Bercioux , P. Lucignano , Rep. Prog. Phys. 2015, 78, 106001. 26406280 10.1088/0034-4885/78/10/106001

[8] F. Sheng , C. Hua , M. Cheng , J. Hu , X. Sun , Q. Tao , H. Lu , Y. Lu , M. Zhong , K. Watanabe , T. Taniguchi , Q. Xia , Z. A. Xu , Y. Zheng , Nature 2021, 593, 56. 33953409 10.1038/s41586-021-03449-8

[9] M. Z. Hasan , C. L. Kane , Rev. Mod. Phys. 2010, 82, 3045.

[10] M. Hong , W. Y. Lyv , M. Li , S. D. Xu , Q. Sun , J. Zou , Z. G. Chen , Joule 2020, 4, 2030.

[11] X. Yang , L. Qiu , Y. Li , H. P. Xue , J. N. Liu , R. Sun , Q. L. Yang , X. S. Gai , Y. S. Wei , A. H. Comstock , D. Sun , X. Q. Zhang , W. He , Y. Hou , Z. H. Cheng , Phys. Rev. Lett. 2023, 131, 186703. 37977650 10.1103/PhysRevLett.131.186703

[12] J. C. Rojas Sanchez , L. Vila , G. Desfonds , S. Gambarelli , J. P. Attane , J. M. De Teresa , C. Magen , A. Fert , Nat. Commun. 2013, 4, 2944. 24343336 10.1038/ncomms3944

[13] P. Noël , F. Trier , L. V. M. Arche , J. Bréhin , D. C. Vaz , V. Garcia , S. Fusil , A. Barthélémy , L. Vila , M. Bibes , J. P. Attané , Nature 2020, 580, 483. 32322081 10.1038/s41586-020-2197-9

[14] Y. Li , Y. Li , P. Li , B. Fang , X. Yang , Y. Wen , D. X. Zheng , C. H. Zhang , X. He , A. Manchon , Z. H. Cheng , X. X. Zhang , Nat. Commun. 2021, 12, 540. 33483483 10.1038/s41467-020-20840-7 PMC7822853

[15] S. Gupta , B. I. Yakobson , J. Am. Chem. Soc. 2021, 143, 3503. 33625213 10.1021/jacs.0c12809

[16] X.‐G. Li , C. Chen , H. Zheng , Y. Zuo , S. P. Ong , Npj Comput. Mater 2020, 6, 70.

[17] V. Sunko , H. Rosner , P. Kushwaha , S. Khim , F. Mazzola , L. Bawden , O. J. Clark , J. M. Riley , D. Kasinathan , M. W. Haverkort , T. K. Kim , M. Hoesch , J. Fujii , I. Vobornik , A. P. Mackenzie , P. D. C. King , Nature 2017, 549, 492. 28959958 10.1038/nature23898

[18] P. D. King , R. C. Hatch , M. Bianchi , R. Ovsyannikov , C. Lupulescu , G. Landolt , B. Slomski , J. H. Dil , D. Guan , J. L. Mi , E. D. Rienks , J. Fink , A. Lindblad , S. Svensson , S. Bao , G. Balakrishnan , B. B. Iversen , J. Osterwalder , W. Eberhardt , F. Baumberger , P. Hofmann , Phys. Rev. Lett. 2011, 107, 096802. 21929260 10.1103/PhysRevLett.107.096802

[19] Q. Song , H. Zhang , T. Su , W. Yuan , Y. Chen , W. Xing , J. Shi , J. Sun , W. Han , Sci. Adv. 2017, 3, 1602312. 10.1126/sciadv.1602312 PMC5357130 28345050

[20] H. Zhang , Y. Ma , H. Zhang , X. Chen , S. Wang , G. Li , Y. Yun , X. Yan , Y. Chen , F. Hu , J. Cai , B. Shen , W. Han , J. Sun , Nano Lett. 2019, 19, 1605. 30715894 10.1021/acs.nanolett.8b04509

[21] K. Hosokawa , M. Yama , M. Matsuo , T. Kato , Phys. Rev. B 2024, 110, 035309.

[22] D. Niesner , M. Wilhelm , I. Levchuk , A. Osvet , S. Shrestha , M. Batentschuk , C. Brabec , T. Fauster , Phys. Rev. Lett. 2016, 117, 126401. 27689285 10.1103/PhysRevLett.117.126401

[23] D. Niesner , M. Hauck , S. Shrestha , I. Levchuk , G. J. Matt , A. Osvet , M. Batentschuk , C. Brabec , H. B. Weber , T. Fauster , Proc. Natl. Acad. Sci. USA 2018, 115, 9509. 30181293 10.1073/pnas.1805422115 PMC6156630

[24] X. Liu , A. Chanana , U. Huynh , F. Xue , P. Haney , S. Blair , X. Jiang , Z. V. Vardeny , Nat. Commun. 2020, 11, 323. 31949152 10.1038/s41467-019-14073-6 PMC6965620

[25] K. Kim , E. Vetter , L. Yan , C. Yang , Z. Q. Wang , R. Sun , Y. Yang , A. H. Comstock , X. Li , J. Zhou , L. F. Zhang , W. You , D. L. Sun , J. Liu , Nat. Mater. 2023, 22, 322. 36781951 10.1038/s41563-023-01473-9

[26] F. Zheng , L. Z. Tan , S. Liu , A. M. Rappe , Nano Lett. 2015, 15, 7794. 26461166 10.1021/acs.nanolett.5b01854

[27] H. M. Yi , L. H. Hu , Y. X. Wang , R. Xiao , J. Q. Cai , D. R. Hickey , C. Y. Dong , Y. F. Zhao , L. J. Zhou , R. X. Zhang , A. R. Richardella , N. Alem , J. A. Robinson , M. H. W. Chan , X. D. Xu , N. Samarth , C. X. Liu , C. Z. Chang , Nat. Mater. 2022, 21, 1366. 36302957 10.1038/s41563-022-01386-z

[28] E. Wang , P. Tang , G. Wan , A. V. Fedorov , I. Miotkowski , Y. P. Chen , W. Duan , S. Zhou , Nano Lett. 2015, 15, 2031. 25710329 10.1021/nl504900s

[29] K. Ishizaka , M. S. Bahramy , H. Murakawa , M. Sakano , T. Shimojima , T. Sonobe , K. Koizumi , S. Shin , H. Miyahara , A. Kimura , K. Miyamoto , T. Okuda , H. Namatame , M. Taniguchi , R. Arita , N. Nagaosa , K. Kobayashi , Y. Murakami , R. Kumai , Y. Kaneko , Y. Onose , Y. Tokura , Nat. Mater. 2011, 10, 521. 21685900 10.1038/nmat3051

[30] M. Liebmann , C. Rinaldi , D. Di Sante , J. Kellner , C. Pauly , R. N. Wang , J. E. Boschker , A. Giussani , S. Bertoli , M. Cantoni , L. Baldrati , M. Asa , I. Vobornik , G. Panaccione , D. Marchenko , J. Sanchez‐Barriga , O. Rader , R. Calarco , S. Picozzi , R. Bertacco , M. Morgenstern , Adv. Mater. 2016, 28, 560. 26599640 10.1002/adma.201503459

[31] L. Bawden , J. M. Riley , C. H. Kim , R. Sankar , E. J. Monkman , D. E. Shai , H. F. I. Wei , E. B. Lochocki , J. W. Wells , W. Meevasana , T. K. Kim , M. Hoesch , Y. Ohtsubo , P. Le Fèvre , C. J. Fennie , K. M. Shen , F. C. Chou , P. D. C. King , Sci. Adv. 2015, 1, 1500495. 10.1126/sciadv.1500495 PMC4643772 26601268

[32] S. W. Cho , Y. W. Lee , S. H. Kim , S. Han , I. Kim , J.‐K. Park , J. Y. Kwak , J. Kim , Y. Jeong , G. W. Hwang , K. S. Lee , S. Park , S. Lee , J. Alloys. Compd. 2023, 957, 170444.

[33] T. Y. Lee , J. Chang , M. C. Hickey , H. C. Koo , H. J. Kim , S. H. Han , J. S. Moodera , Appl. Phys. Lett. 2011, 98, 202504.

[34] K. He , T. Hirahara , T. Okuda , S. Hasegawa , A. Kakizaki , I. Matsuda , Phys. Rev. Lett. 2008, 101, 107604. 18851258 10.1103/PhysRevLett.101.107604

[35] Y. Zhang , K. He , C.‐Z. Chang , C.‐L. Song , L.‐L. Wang , X. Chen , J.‐F. Jia , Z. Fang , X. Dai , W.‐Y. Shan , S.‐Q. Shen , Q. Niu , X.‐L. Qi , S.‐C. Zhang , X.‐C. Ma , Q.‐K. Xue , Nat. Phys. 2010, 6, 584.

[36] S. Mathias , A. Ruffing , F. Deicke , M. Wiesenmayer , I. Sakar , G. Bihlmayer , E. V. Chulkov , Y. M. Koroteev , P. M. Echenique , M. Bauer , M. Aeschlimann , Phys. Rev. Lett. 2010, 104, 066802. 20366845 10.1103/PhysRevLett.104.066802

[37] G. Wang , B. L. Liu , A. Balocchi , P. Renucci , C. R. Zhu , T. Amand , C. Fontaine , X. Marie , Nat. Commun. 2013, 4, 2372. 24052071 10.1038/ncomms3372 PMC3791469

[38] S.‐J. Gong , C.‐G. Duan , Y. Zhu , Z.‐Q. Zhu , J.‐H. Chu , Phys. Rev. B 2013, 87, 035403.

[39] K. Premasiri , S. K. Radha , S. Sucharitakul , U. R. Kumar , R. Sankar , F. C. Chou , Y. T. Chen , X. P. A. Gao , Nano Lett. 2018, 18, 4403. 29860844 10.1021/acs.nanolett.8b01462

[40] X. Yang , X. M. Li , Y. Li , Y. Li , R. Sun , J. N. Liu , X. Bai , N. Li , Z. K. Xie , L. Su , Z. Z. Gong , X. Q. Zhang , W. He , Z. Cheng , Nano Lett. 2021, 21, 77. 33263408 10.1021/acs.nanolett.0c03161

[41] H. Ryu , J.‐M. Lihm , J. Cha , B. Kim , B. S. Kim , W. Kyung , I. Song , Y. Kim , G. Han , J. Denlinger , I. Chung , C.‐H. Park , S. R. Park , C. Kim , Phys. Rev. B 2021, 103, 245113.

[42] G. Kremer , J. Maklar , L. Nicolai , C. W. Nicholson , C. Yue , C. Silva , P. Werner , J. H. Dil , J. Krempasky , G. Springholz , R. Ernstorfer , J. Minar , L. Rettig , C. Monney , Nat. Commun. 2022, 13, 6396. 36302853 10.1038/s41467-022-33978-3 PMC9613697

[43] Q. Peng , Y. Lei , X. Deng , J. Deng , G. Wu , J. Li , C. He , J. Zhong , Physica E: Low Dimens. Syst. Nanostruct. 2022, 135, 114944.

[44] J. J. Zhou , W. Feng , Y. Zhang , S. A. Yang , Y. Yao , Sci. Rep. 2014, 4, 3841. 24452501 10.1038/srep03841 PMC3899590

[45] Z. Liang , J. Zhang , C. Hua , Y. Wang , F. Song , Phys. Rev. B 2024, 110, 085110.

[46] W. M. Xue , J. Li , X. Y. Peng , C. Y. He , T. Ouyang , X. Qi , C. X. Zhang , C. B. Luo , J. Deng , Q. Peng , S. F. Zhang , C. Tang , J. X. Zhong , J. Electron. Mater. 2022, 51, 5142.

[47] L. Petersen , P. Hedegård , Surf. Sci. 2000, 459, 49.

[48] B. Croes , A. Llopez , C. Tagne‐Kaegom , B. Tegomo‐Chiogo , B. Kierren , P. Müller , S. Curiotto , P. Le Fèvre , F. Bertran , A. Saúl , Y. Fagot‐Revurat , F. Leroy , F. Cheynis , Nano Lett. 2024, 24, 13224. 39401414 10.1021/acs.nanolett.4c03281

[49] D. P. A. Holgado , K. Bolaños , S. de Castro , H. S. A. Monteiro , F. S. Pena , A. K. Okazaki , C. I. Fornari , P. H. O. Rappl , E. Abramof , D. A. W. Soares , M. L. Peres , Appl. Phys. Lett. 2020, 117, 102108.

[50] D. Di Sante , P. Barone , R. Bertacco , S. Picozzi , Adv. Mater. 2013, 25, 509. 23070981 10.1002/adma.201203199

[51] C. R. Ast , J. Henk , A. Ernst , L. Moreschini , M. C. Falub , D. Pacile , P. Bruno , K. Kern , M. Grioni , Phys. Rev. Lett. 2007, 98, 186807. 17501597 10.1103/PhysRevLett.98.186807

[52] H. C. Koo , J. H. Kwon , J. Eom , J. Chang , S. H. Han , M. Johnson , Science 2009, 325, 1515. 19762637 10.1126/science.1173667

[53] M. Sakano , M. S. Bahramy , A. Katayama , T. Shimojima , H. Murakawa , Y. Kaneko , W. Malaeb , S. Shin , K. Ono , H. Kumigashira , R. Arita , N. Nagaosa , H. Y. Hwang , Y. Tokura , K. Ishizaka , Phys. Rev. Lett. 2013, 110, 107204. 23521291 10.1103/PhysRevLett.110.107204

[54] T. Ideue , K. Hamamoto , S. Koshikawa , M. Ezawa , S. Shimizu , Y. Kaneko , Y. Tokura , N. Nagaosa , Y. Iwasa , Nat. Phys. 2017, 13, 578.

[55] Y. M. Koroteev , G. Bihlmayer , J. E. Gayone , E. V. Chulkov , S. Blugel , P. M. Echenique , P. Hofmann , Phys. Rev. Lett. 2004, 93, 046403. 15323779 10.1103/PhysRevLett.93.046403

[56] Z. Zhong , L. Si , Q. Zhang , W. G. Yin , S. Yunoki , K. Held , Adv. Mater. Interfaces 2015, 2, 1400445.

[57] N. Bansal , Y. S. Kim , M. Brahlek , E. Edrey , S. Oh , Phys. Rev. Lett. 2012, 109, 116804. 23005664 10.1103/PhysRevLett.109.116804

[58] H. Sang , W. Wang , Z. Wang , M. Hong , C. Zhang , S. Xie , H. Ge , F. Yan , Z. Wang , Y. Ouyang , Y. Liu , J. Wu , W. Liu , X. Tang , Adv. Funct. Mater. 2022, 33, 2210213.

[59] J. G. Analytis , J.‐H. Chu , Y. Chen , F. Corredor , R. D. McDonald , Z. X. Shen , I. R. Fisher , Phys. Rev. B 2010, 81, 205407.

[60] T. Sato , K. Sugawara , T. Kato , Y. Nakata , S. Souma , K. Yamauchi , T. Oguchi , T. Takahashi , T. Sato , ACS Appl. Electron. Mater. 2021, 3, 1080.

[61] M. Vagadia , J. Sahoo , A. Kumar , S. Sardar , T. M. Tank , D. S. Rana , Phys. Rev. B 2023, 107, 064420.

[62] P. Nukala , M. Ren , R. Agarwal , J. Berger , G. Liu , A. T. Johnson , R. Agarwal , Nat. Commun. 2017, 8, 15033. 28401949 10.1038/ncomms15033 PMC5394341

[63] S. Varotto , L. Nessi , S. Cecchi , J. Sławińska , P. Noël , S. Petrò , F. Fagiani , A. Novati , M. Cantoni , D. Petti , E. Albisetti , M. Costa , R. Calarco , M. Buongiorno Nardelli , M. Bibes , S. Picozzi , J.‐P. Attané , L. Vila , R. Bertacco , C. Rinaldi , Nat. Electron. 2021, 4, 740.

[64] Y. Y. Li , G. Wang , X. G. Zhu , M. H. Liu , C. Ye , X. Chen , Y. Y. Wang , K. He , L. L. Wang , X. C. Ma , H. J. Zhang , X. Dai , Z. Fang , X. C. Xie , Y. Liu , X. L. Qi , J. F. Jia , S. C. Zhang , Q. K. Xue , Adv. Mater. 2010, 22, 4002. 20648518 10.1002/adma.201000368

[65] G. Kresse , J. Furthmüller , Phys. Rev. B 1996, 54, 11169. 10.1103/physrevb.54.11169 9984901

[66] J. P. P. K. Burke , M. Ernzerhof , Phys. Rev. Lett. 1996, 77, 3865. 10062328 10.1103/PhysRevLett.77.3865

[67] P. E. Blöchl , Phys. Rev. B 1994, 50, 17953. 10.1103/physrevb.50.17953 9976227

[68] G. Kresse , D. Joubert , Phys. Rev. B 1999, 59, 1758.

[69] G. Stefan , J. Comput. Chem. 2006, 27, 1787. 16955487 10.1002/jcc.20495

