# Supplementary material for: Maximal Rashba Splitting in GeTe/Bi2Te3 Heterostructures via Strong Band Bending
Source: Adv Sci (Weinh). 2025 Nov 7;13(5):e13673. doi: 10.1002/advs.202513673 (PMC12849887; doi:10.1002/advs.202513673)
Supplement: Supplementary file 1 — Supporting Information [file ADVS-13-e13673-s001.pdf]

Supplemental Information for
Maximal Rashba Splitting in GeTe/Bi_2Te_3 heterostructures via strong band bending

Qing-Lin Yang[1,3,7], Xu Yang[1,6,7], Jia-wan Li[2,7], Yan Li[1], Jin Tang[4,5], Hai-Feng Du[4,5], Zi-zhao Gong[1], Hao-Pu Xue[1,3], Jia-Nan Liu[1,3], Zhuo Deng[1,3], Peng-Tao Yang[1,3], Xiang-Qun Zhang[1], Wei He[1], Yusheng Hou[2]*, and Zhao-Hua Cheng[1,3,6,]*

[1]Beijing National Laboratory for Condensed Matter Physics, Institute of Physics, Chinese Academy of Sciences, Beijing 100190, China
[2]Guangdong Provincial Key Laboratory of Magnetoelectric Physics and Devices, Center for Neutron Science and Technology, School of Physics, Sun Yat-Sen University, Guangzhou, 510275, China
[3]School of Physical Sciences, University of Chinese Academy of Sciences, Beijing 100049, China
[4]Anhui Province Key Laboratory of Condensed Matter Physics at Extreme Conditions, High Magnetic Field Laboratory of the Chinese Academy of Sciences, and University of Science and Technology of China, Hefei 230031, China;
[5]Institute of Physical Science and Information Technology, Anhui University, Hefei 230601, China;
[6]Songshan Lake Materials Laboratory, Dongguan, Guangdong 523808, China
[7]These authors contributed equally: Qing-Lin Yang, Xu Yang and Jia-wan Li
*Corresponding authors: zhcheng@iphy.ac.cn or houysh@mail.sysu.edu.cn

Content

S1. Structural characterization of GeTe/Bi_2Te_3 heterostructures.

S2. MDCs of ARPES spectrum for GeTe (1 nm)/Bi_2Te_3 (1 QL) heterostructure and the fitting results of the MDC.

S3. Temperature-dependence of longitudinal resistivity for GeTe/Bi_2Te_3 heterostructures.

S4. Magneto-conductance of GeTe/Bi_2Te_3 heterostructures.

S5. Atomic and electronic structures of GeTe/Bi_2Te_3 (1 QL) heterostructures with and without alpha surface reconstruction.

S6. The role of SOC in band structures.

S7. ARPES spectra of GeTe and Bi_2Te_3.

S8. ARPES results of GeTe (x nm)/Bi_2Te_3 (10 QL) heterostructures and its second derivative.

S9. ARPES results of GeTe (x nm)/Bi_2Te_3 (10 QL) heterostructures with GeTe thickness, *x*= 5.0, 7.0, 8.0, and 10.0 nm.

S10. Integral EDC curves of GeTe (*x* nm)/Bi_2Te_3 (10 QL) heterostructures with different GeTe thickness, *x*.

S11. ARPES results of GeTe (*x* nm)/Bi_2Te_3 (1 QL) heterostructures.

S1. Structural characterization of GeTe/Bi_2Te_3 heterostructures

The GeTe/Bi_2Te_3 heterostructures were prepared as shown in the lower panel of Figure S1(a). To confirm the quality of the heterostructures, the epitaxial growth is characterized by cross-section high-resolution transmission electron microscopy (HRTEM), reflection high-energy electron diffraction (RHEED), X-ray diffraction (XRD), as well as reciprocal space mapping (RSM). Figure S1(b) displays a representative HRTEM image of a GeTe (30 nm)/Bi_2Te_3 (10 QL)/Si (111) heterostructure. This result indicates the heterostructure has a sharp interface, with both the GeTe and Bi_2Te_3 layers being of high quality. The RHEED spots of Bi_2Te_3 and GeTe demonstrate that the direction of electron beam incidence is along $\bar{\Gamma} - \bar{\mathrm{M}}$, the red dashed lines represent the center of RHEED streaks, implying the epitaxial growth on Bi_2Te_3 (Figure S1(c)). Sharp XRD peaks of GeTe (000l) and Bi_2Te_3(000l) are evident (Figure S1(d)). Moreover, the reciprocal space mapping (RSM) of the heterostructure is measured around Si (1 1 -1). The iso-intensity contours are plotted in reciprocal space co-ordinates in Figure S1(e). The misalignment and broadening of substrate Si (1 1 -1), GeTe (0 1 1) and Bi_2Te_3 (0 1 3) nodes along the Q_x direction the due to the partial relaxation and mosaicity. The in-plane strains of GeTe and Bi_2Te_3 is up to 5%, consistent with the reports[1]. Moreover, the high-angle annular dark field (HAADF) image of GeTe(30nm)/Bi_2Te_3(10 QL) heterostructure is shown in Figure S1(f), the element Bi and Ge show a sharp interface, means there is no obvious diffusion. All these results suggest that GeTe can well epitaxially grow on Bi_2Te_3.

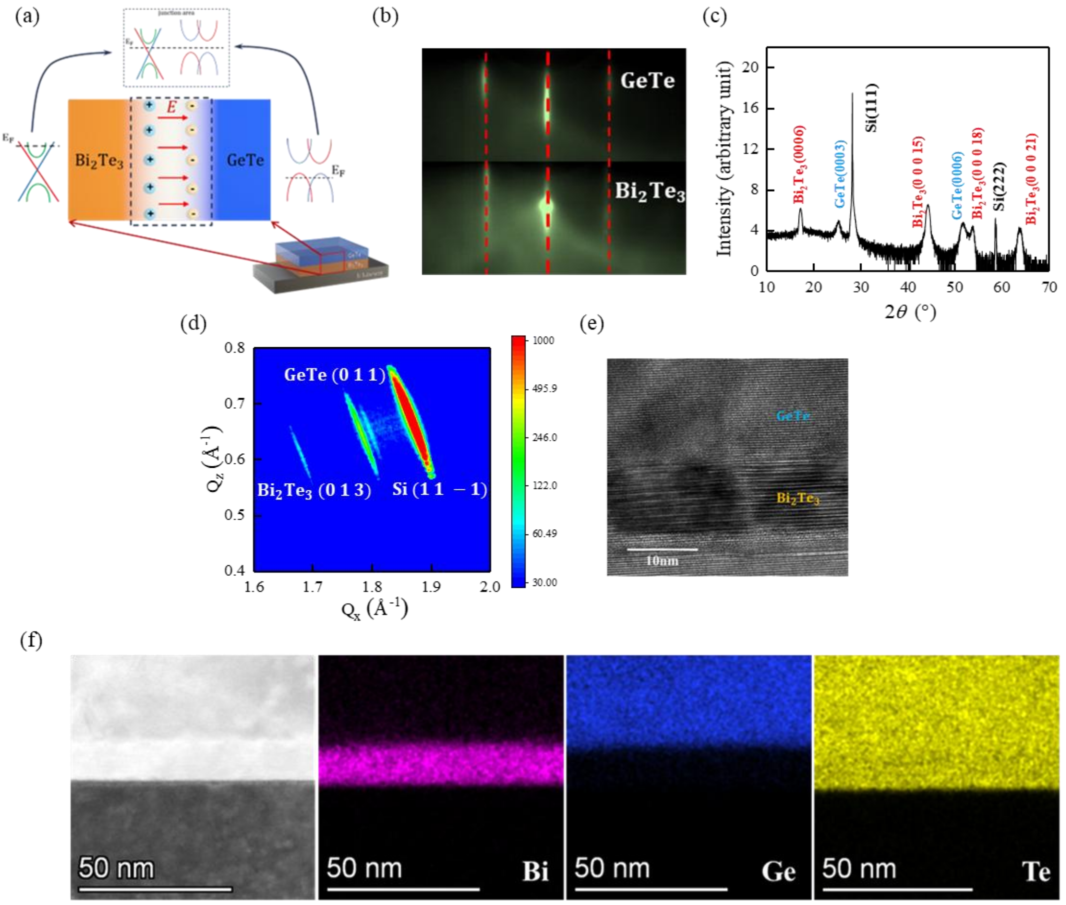

Figure. S1 **(a)** Schematic of GeTe/Bi_2Te_3 heterostructure. **(b)** Cross-section HRTEM image of GeTe(30nm)//Bi_2Te_3(10 QL) heterostructure. **(c)** RHEED patterns of GeTe/Bi_2Te_3(0001) (upper panel) and GeTe (0001) (lower panel) along $[\bar{1}100]$. **(d)** XRD pattern of GeTe/Bi_2Te_3 (000l)/Si (111). **(e)** Reciprocal space map of GeTe/Bi_2Te_3 heterostructure around Si (1 1 -1). **(f)** The HAADF image of GeTe(30nm)/Bi_2Te_3(10 QL) heterostructure cross section and the elemental compositions of Bi, Ge and Te are mapped by pink, blue and yellow, respectively.

S2. MDCs of the ARPES spectrum for GeTe (1 nm)/Bi_2Te_3 (1 QL) heterostructures and the fitting results of the MDC.

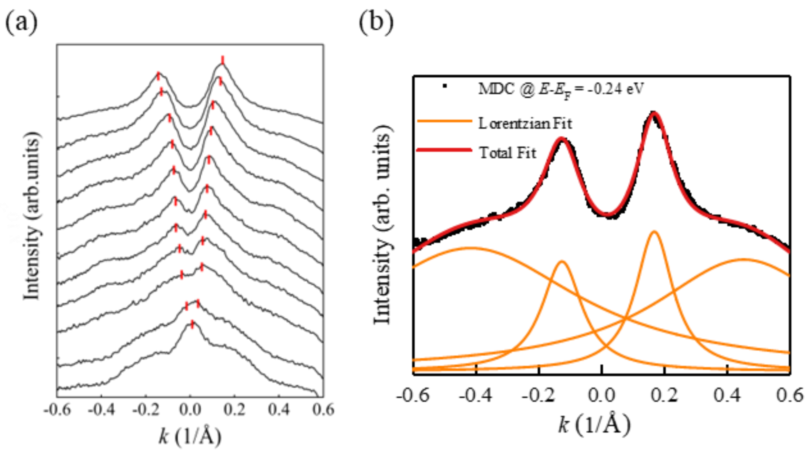

Figure S2 (a) MDCs of the ARPES spectrum for GeTe (1.0 nm)/Bi_2Te_3 (1 QL) /Si (111) heterostructure. The red tick marks indicate the peaks positions. **(b)** Fitting results of the MDC. The black line represents the MDC, the orange lines represent the muti-Lorentzian fitting of the individual peaks, and the red line represent the total fitting line of the MDC.

S3. Temperature-dependence of longitudinal resistivity for GeTe/Bi_2Te_3 heterostructures.

We measured the temperature-dependent longitudinal resistivity of GeTe (3 nm)/Bi_2Te_3 (2 QL) and GeTe (30 nm) Bi_2Te_3 (10 QL) heterostructures (Figure S4). For the GeTe (30 nm)/Bi_2Te_3 (10 QL) heterostructure, the longitudinal resistivity increases with temperature (red line), suggesting metallic behavior. The residual resistivity ratio (RRR, $\rho_{300\mathrm{K}}/\rho_{2\mathrm{K}}$) for this heterostructure is 1.54, comparable to values reported for GeTe films[2, 3, 4], indicating the high quality of the samples. In contrast, the longitudinal resistivity of the GeTe (3 nm)/Bi_2Te_3 (2 QL) heterostructure decreases with increasing temperature (black line), characteristic of semiconductor behavior. Notably, the longitudinal resistivity of the GeTe (3 nm)/Bi_2Te_3 (2 QL) heterostructure is significantly higher than that of the GeTe (30 nm)/Bi_2Te_3 (10 QL) heterostructure. This higher resistivity and semiconductor behavior suggest a reduction in carrier concentration due to charge transfer at the interface. For the GeTe (30 nm)/Bi_2Te_3 (10 QL) heterostructure, charge transfer still occurs but is confined to a small region near the interface, allowing the longitudinal resistivity to resemble that of normal GeTe or Bi_2Te_3, which are metallic. This result is consistent with ARPES measurements. Specifically, for the GeTe (30 nm)/Bi_2Te_3 (10 QL) heterostructure, the Fermi level of GeTe is located in the valence band, while that of Bi_2Te_3 is in the conduction band (Fig. S6), indicating metallic characteristics. In contrast, as shown in Figure 1(c), when the GeTe thickness is reduced in GeTe/Bi_2Te_3 heterostructures, the Fermi level lies within the band gap, leading to semiconductor behavior.

To evaluate surface oxidation, we performed XPS measurements on GeTe (3 nm)/Bi_2Te_3 (2 QL) after air exposure (Fig. S3(b)). The spectrum (28–43 eV binding energy window) shows the dominated Te 4d and Ge 3d peaks consistent with pristine GeTe/Bi_2Te_3 interfaces. Critically, the oxidized component remains negligible relative to principal peaks. [5] While surface oxidation *could* alter electrical properties in ultrathin films (~nm scale), our magnetoconductance measurements definitively exclude this scenario. The observed weak antilocalization—manifested as magnetic-

field-dependent conductance—directly demonstrates Rashba spin-orbit coupling at the interface. This quantum transport signature is inconsistent with oxidation-induced disorder and instead confirms the intrinsic SOC origin.

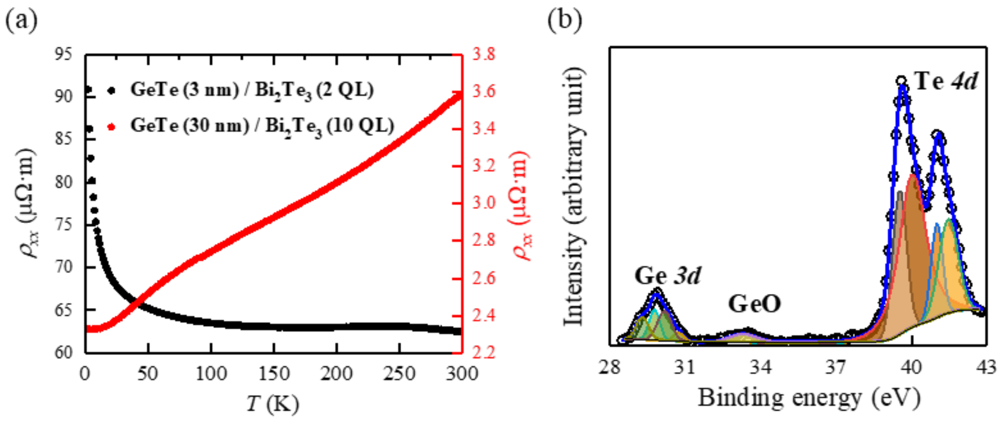

Figure. S3 (a) Temperature-dependence of longitudinal resistivity GeTe (3.0 nm)/Bi_2Te_3 (2 QL) and GeTe (30.0 nm)/Bi_2Te_3 (10 QL) heterostructures. **(b)** XPS measurements of GeTe (3 nm)/Bi_2Te_3(2 QL) after exposed to air.

S4. Magneto-conductance of GeTe/Bi_2Te_3 heterostructures.

The Rashba spin-orbit coupling (SOC) effect can be investigated through magneto-conductance measurements. Strong SOC leads to weak antilocalization (WAL) [6, 7]. Magneto-conductance measurements were performed on GeTe (3 nm)/Bi_2Te_3 (2 QL) and GeTe (30 nm)/Bi_2Te_3 (10 QL) heterostructures, with the results presented in Figure S5. As shown in Figure S5(a) and Figure S5(c), both heterostructures exhibit characteristics of weak antilocalization, indicating strong spin-orbit coupling in the heterostructures. Additionally, the magneto-conductance data were fitted using the Hikami-Larkin-Nagaoka (HLN) equation[7]:

$$\Delta\sigma(B) = \frac{\alpha \mathrm{e}^2}{2\pi^2\hbar}\left[\ln\left(\frac{B_\phi}{B}\right) - \psi\left(\frac{1}{2}+\frac{B_\phi}{B}\right)\right]$$

$$-\frac{3\alpha \mathrm{e}^2}{2\pi^2\hbar}\left[\ln\left(\frac{\frac{4}{3}B_{\mathrm{so}}+B_\phi}{B}\right) - \psi\left(\frac{1}{2}+\frac{\frac{4}{3}B_{\mathrm{so}}+B_\phi}{B}\right)\right] \qquad (2)$$

where $\psi(x)$ is the digamma function and $B_i = \frac{\hbar}{4el_i^2}$ $(i = \phi,\ \mathrm{so})$. l_ϕ is the phase coherence length and l_{so} is the spin–orbit relaxation length. The red line in Figure S5(b) and Figure S5(d) represents the fitting line and the parameter of GeTe (3 nm)/Bi_2Te_3 (2 QL) heterostructure is $\alpha = 0.115 \pm 0.003$, $l_\phi = (48.5 \pm 0.8)$ nm and $l_{so} = (5.3 \pm 3.0)$ nm. For the GeTe (30 nm)/Bi_2Te_3 (10 QL) heterostructure, he corresponding values are $\alpha = 1.03 \pm 0.02$, $l_\phi = (82.2 \pm 1.9)$ nm and $l_{so} = (8.3 \pm 2.2)$ nm. Importantly, the value of l_{so} is inversely proportional to the strength of Rashba SOC. The l_{so} of GeTe (3 nm)/Bi_2Te_3 (2 QL) heterostructure is smaller than that of the GeTe (30 nm)/Bi_2Te_3 (10 QL) heterostructure, indicating that the Rashba SOC is enhanced at the interface of the heterostructures.

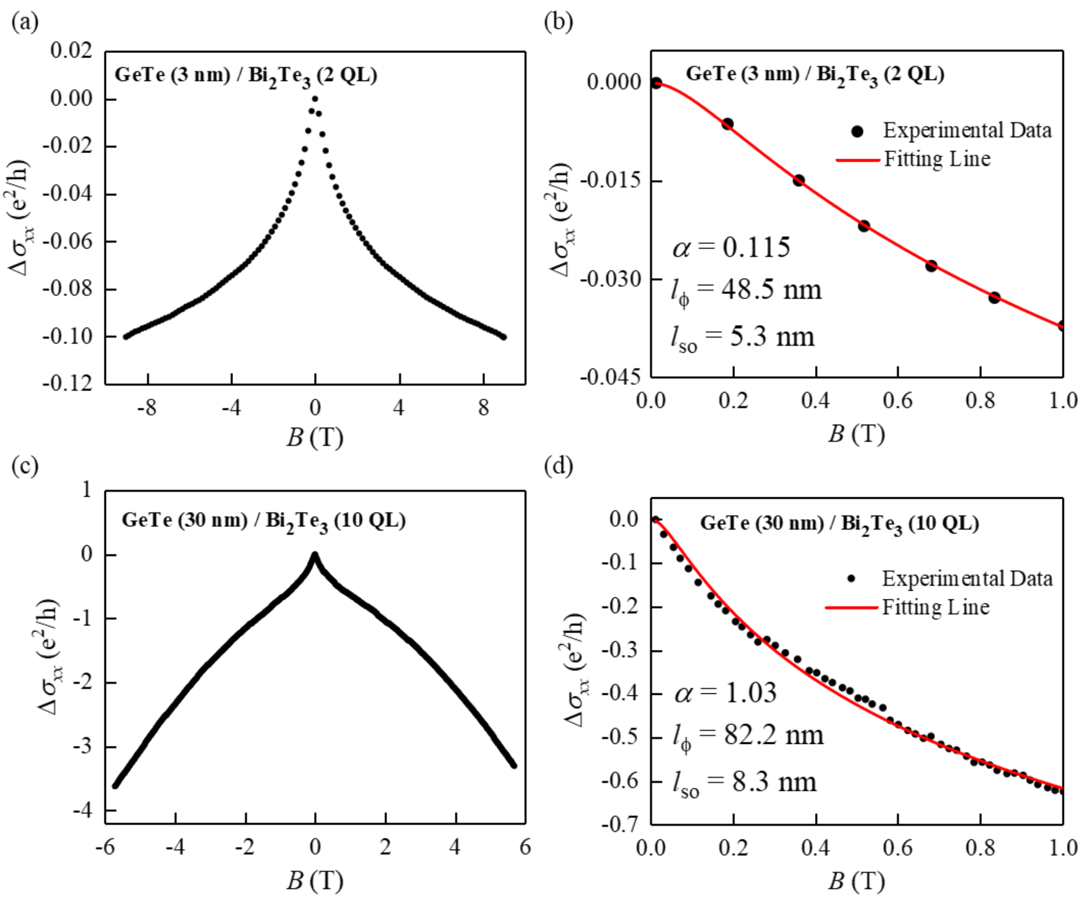

Figure. S4 (a) Magneto-conductance of the GeTe (3 nm)/Bi_2Te_3 (2 QL) heterostructure. and GeTe (30 nm)/ Bi_2Te_3 (10 QL) heterostructure **(b)** Fitting of the magneto-conductance response in (a) using the HLN equation. **(c)** Magneto-conductance of the GeTe (30 nm)/ Bi_2Te_3 (10 QL) heterostructure. **(d)** Fitting of the magneto-conductance response in (c) using the HLN equation.

S5. Atomic and electronic structures of $GeTe/Bi_2Te_3$ (1 QL) heterostructures with and without alpha surface reconstruction.

To assess the impact of surface reconstruction on the $GeTe/Bi_2Te_3$ (1 QL) interface, we compare two representative models: one without surface reconstruction and one with the alpha reconstruction featuring a Ge vacancy [8]. The optimized atomic structures of these two models are shown in Figure S5(a–d). Without surface reconstruction, the optimized interface forms direct bonding between Ge and Te atoms, as highlighted in the enlarged view of Figure S5b. In contrast, the alpha reconstructed surface further induces significant displacement of the interfacial Bi atoms, as shown in the enlarged view of Figure S5d.

These structural differences strongly influence the electronic properties. As shown in Figure S5(e, f), the unreconstructed model remains metallic, whereas the alpha reconstructed model exhibits a band gap of approximately 40 meV along the $\overline{\mathrm{M}}-\overline{\Gamma}-\overline{\mathrm{M}}$ path. Therefore, the experimentally observed insulating behavior may be due to atomic rearrangements at the interface induced by possible GeTe surface reconstruction. Although surface reconstruction leads to band gap opening, the overall spin-resolved band structures (S_y component) still retain clear Rashba-type features. In particular, the alpha reconstructed model preserves the characteristic Rashba spin-split bands near the Γ point, and the band dispersion remains qualitatively similar to that of unreconstructed model. Notably, the Rashba band structure of the alpha reconstructed model is consistent with experimental observations in terms of band profiles.

To further confirm the Rashba nature of these states, we analyze the constant energy contours and spin textures of the reconstructed model at energies of –0.10 eV and –0.15 eV below the Fermi level (Fig. S5g, h). The results show well-defined spin-momentum locking: at –0.10 eV (Fig. S5g, above the Rashba crossing point), the spin orientation forms a uniform pattern, while at –0.15 eV (Fig. S5h, below the crossing point) the spin texture exhibits an opposite helicity. These results provide strong evidence that the reconstructed model still hosts Rashba-type spin splitting.

We obtain that the calculated Rashba parameter for the reconstructed model is 3.51

eV·Å, which is somewhat smaller than the experimental value of 6.72 eV·Å. This difference may originate from additional interfacial complexities in the experimental samples, such as further atomic disorder, strain effects, or interactions beyond those included in our model. Nonetheless, our calculations capture the essential Rashba physics as observed in our experiments.

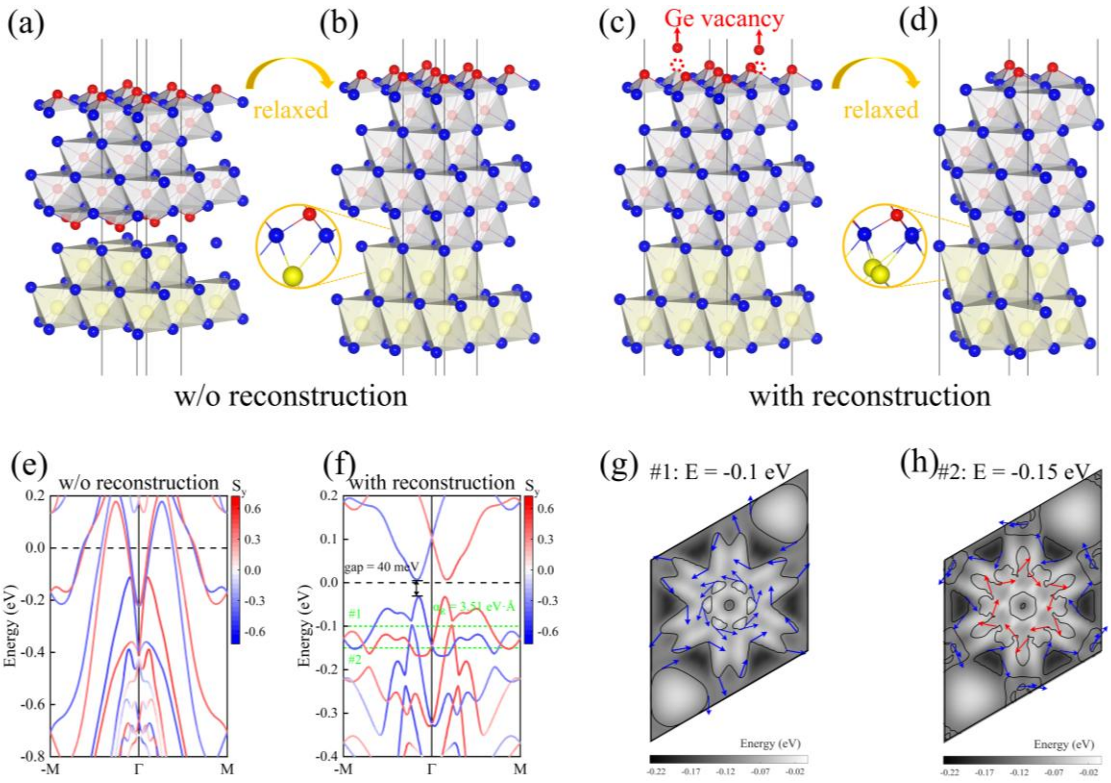

Figure. S5 Atomic structures of GeTe/Bi_2Te_3 (1 QL): **(a)** unrelaxed and **(b)** optimized structural models without surface reconstruction; **(c)** unrelaxed and **(d)** optimized structural models with the alpha reconstruction. The insets in (b) and (d) highlight the optimized interfacial regions, showing direct Ge–Te bonding without reconstruction (b) and significant Bi displacement in the alpha reconstructed model (d). **(e, f)** Spin-resolved band structures (S_y component) without (e) and with (f) alpha reconstruction. The Rashba crossing point is located at –0.144 eV below the Fermi level. **(g, h)** Constant energy contours and corresponding spin textures of the Rashba bands at (g) –0.10 eV (green dashed line #1 in f) and (h) –0.15 eV (green dashed line #2 in f), respectively. Colored arrows indicate the spin orientation and polarization magnitude.

S6. The role of SOC in band structures.

Additionally, we also examine the role of SOC in the emergence of Rashba splitting by performing a series of DFT calculations, in which SOC is selectively turned on or off in the GeTe and Bi_2Te_3 layers for both the unreconstructed and the alpha reconstructed structural models. Our restuls are shown in Figure S6. For the unreconstructed structure [Figure S6(a–d)], when SOC is included in both GeTe and Bi_2Te_3 layers, a pronounced Rashba spin splitting emerges near the Γ point with opposite spin helicities (same as Figure 2 in the main text). When SOC is switched on only in Bi_2Te_3 or only in GeTe, a much weaker splitting remains owing to interfacial hybridization. If turning off SOC in both Bi_2Te_3 and GeTe layers, the spin textures are completely degenerated and no spin splitting occurs. For the alpha reconstructed structure model [Figure S6(e–h)], the reconstruction slightly alters the band dispersion and opens a band gap along $\overline{\mathrm{M}} - \overline{\Gamma} - \overline{\mathrm{M}}$. Most importantly, the Rashba splitting near Γ and the associated helical spin texture persist when the SOC of both Bi_2Te_3 and GeTe is included, whereas they disappear when switching off SOC. Overall, these results confirm that SOC is essential for producing the experimentally observed Rashba splitting, which manifests under the interfacial inversion-symmetry-breaking environment of the GeTe/Bi_2Te_3 heterostructure.

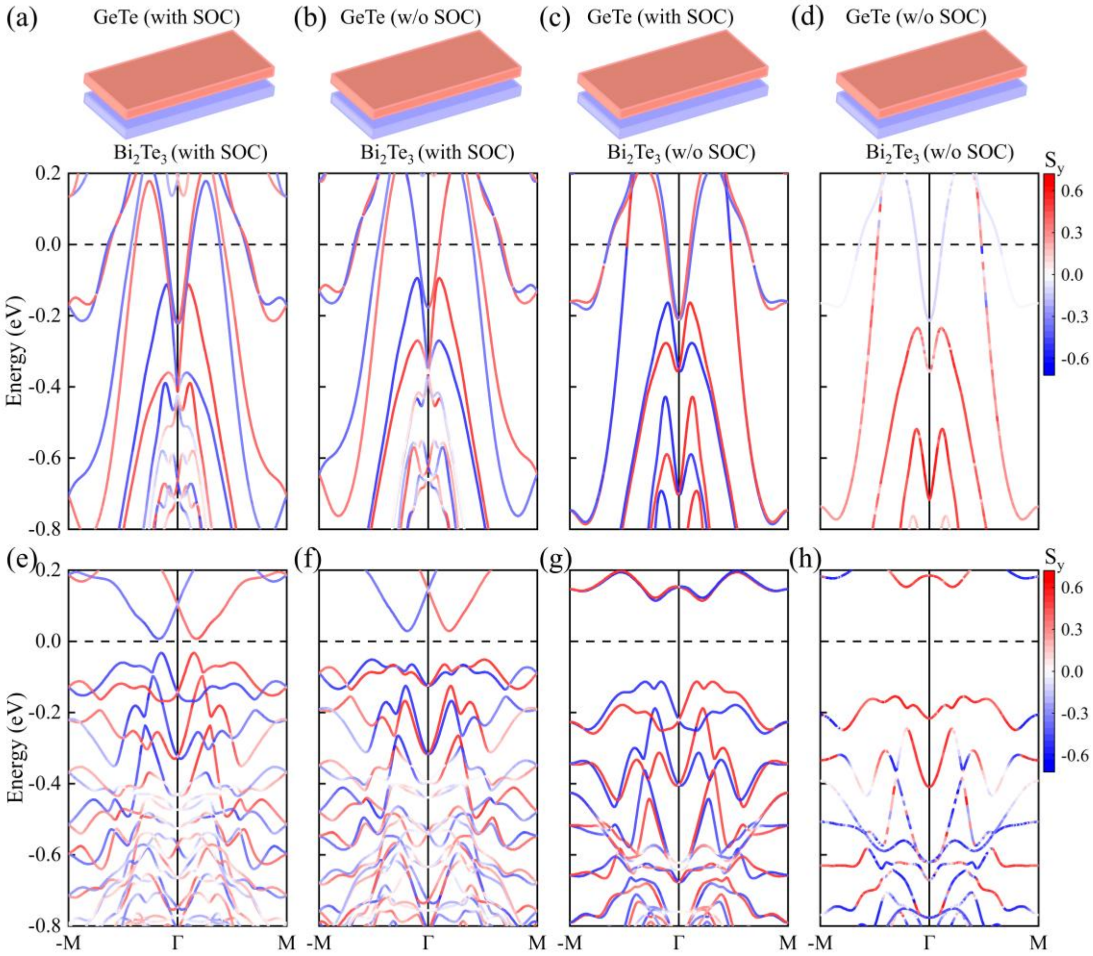

Figure S6 Spin-resolved electronic band structures (S_y component) of the GeTe/Bi_2Te_3 (1 QL) heterostructure along $\bar{M} - \bar{\Gamma} - \bar{M}$, illustrating the effect of spin–orbit coupling (SOC). **(a–d)** show the unreconstructed model corresponding to the main-text Figure 2(a), while **(e–h)** display the alpha reconstructed model described in Supplementary Figure S5(c, d). **(a, e)** Both Bi_2Te_3 and GeTe layers include SOC; **(b, f)** only Bi_2Te_3 has SOC; **(c, g)** only GeTe has SOC; **(d, h)** both layers are non-SOC. The color scale represents the S_y spin component.

S7. ARPES spectra of GeTe and Bi_2Te_3

The opposite carrier types of GeTe and Bi_2Te_3 are key for creating strong charge transfer in GeTe/Bi_2Te_3 heterostructures, as illustrated in the upper panel of Figure S1(a). To investigate the charge transfer, the electronic structure of GeTe/Si (111) and Bi_2Te_3/Si (111) were measured by *in-situ* ARPES (Supplement information Figure S6(a-b)). ARPES spectra demonstrate that GeTe and Bi_2Te_3 have *p*- and *n*-type carriers, respectively. In addition, the band dispersion of GeTe (30 nm) indicates distinct surface states (SS) (green dotted lines) and bulk states (BS) (red dashed lines) along the direction $\overline{\mathrm{K}}-\overline{\Gamma}-\overline{\mathrm{K}}$ (Supplement information Figure S7(a)). In contrast, the band dispersion of Bi_2Te_3 (10 QL) exhibits a topologically protected Dirac cone with linear dispersion, and the Dirac cone point (DP) at about 200 meV below the Fermi level (Supplement information Figure S6(b)). These results are in good agreement with previous reports [9, 10, 11].

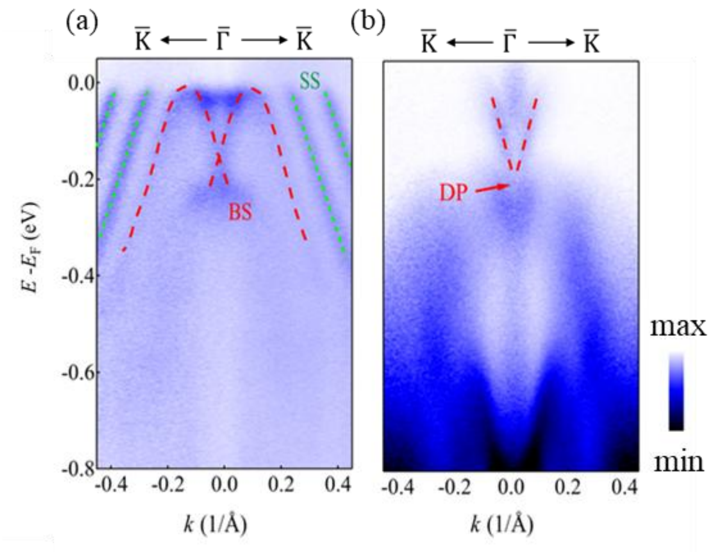

Figure S7 (a) ARPES spectrum of GeTe (30.0 nm). **(b)** ARPES spectrum of Bi_2Te_3 (10 QL)

S8. ARPES results of GeTe (x nm)/Bi_2Te_3 (10 QL) heterostructures and their second derivative.

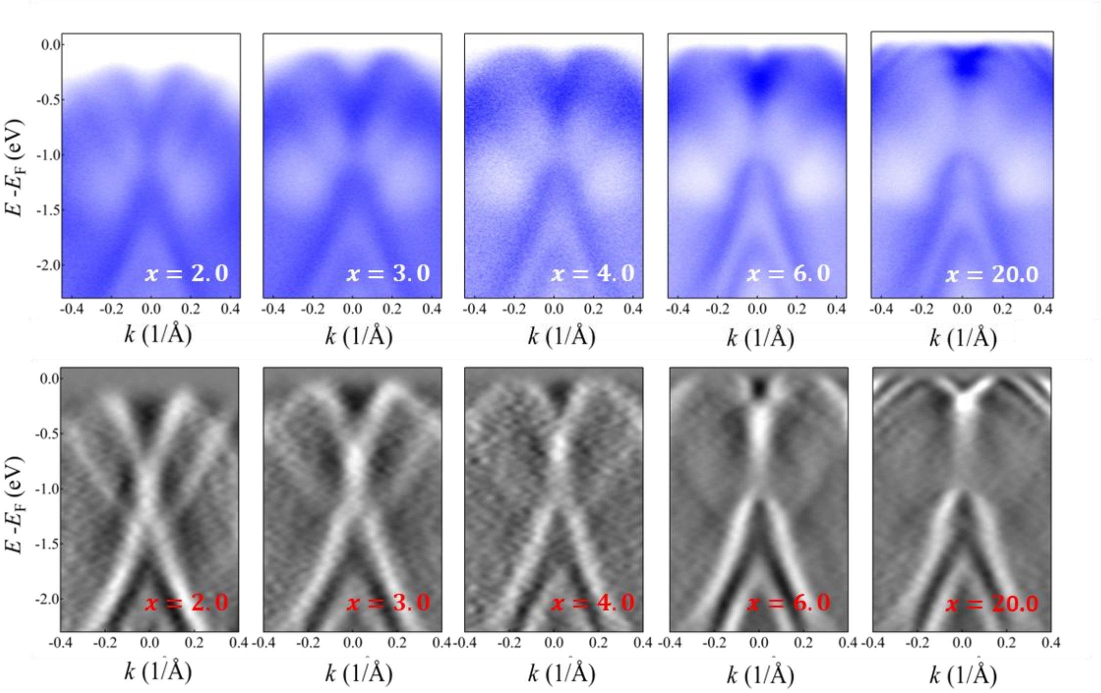

Figure S8 The ARPES spectrums of GeTe (x nm)/Bi_2Te_3 (10 QL) heterostructures and their second derivative.

S9. ARPES results of GeTe (x nm)/Bi_2Te_3 (10 QL) heterostructures with GeTe thickness x= 5.0, 7.0, 8.0, and 10.0 nm.

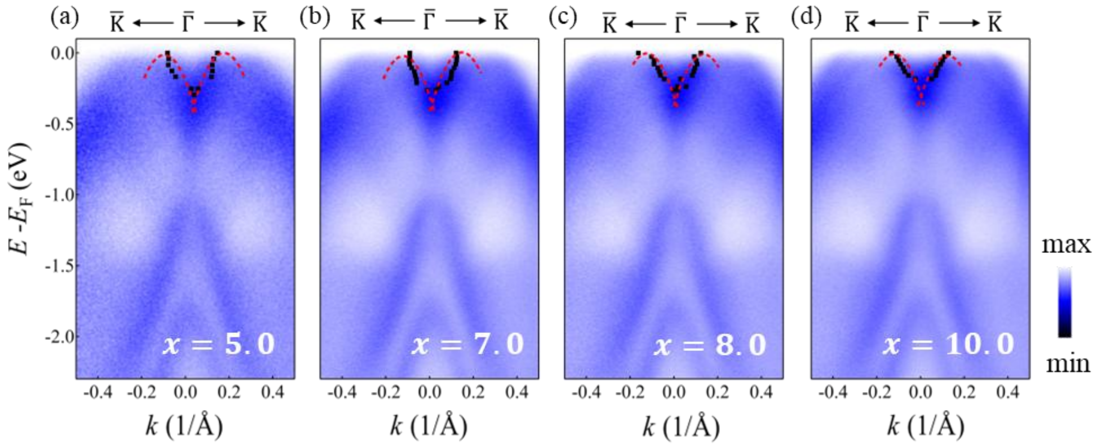

Figure S9 (a-d) ARPES spectrum of GeTe (x nm)/Bi_2Te_3 (10 QL) heterostructures with GeTe thickness x= 5.0, 7.0, 8.0, and 10.0 nm, respectively. The black dots indicate the peak positions of the momentum distribution curves (MDCs). The red dashed lines are the fitted lines for Rashba bands.

S10. Integral EDC curves of GeTe (x nm)/Bi_2Te_3 (10 QL) heterostructures with different GeTe thickness, x.

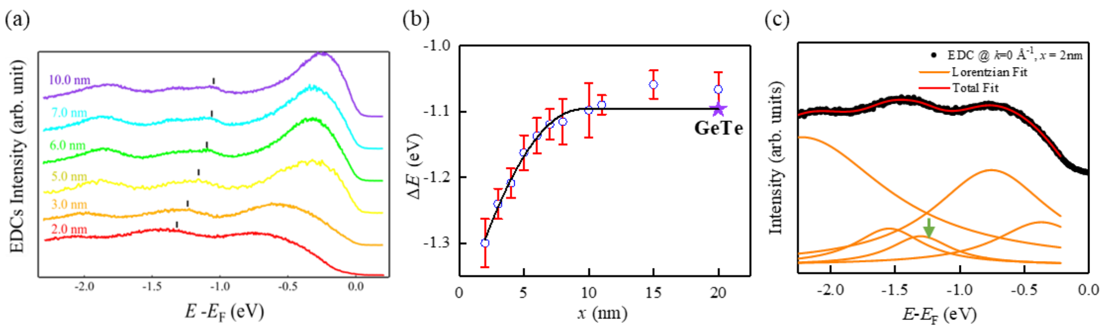

Figure S10 (a) Integral EDC curves of GeTe (x nm)/Bi_2Te_3 (10 QL) heterostructures with different GeTe thickness, x. **(b)** GeTe thickness dependence of Fermi levels of GeTe (x nm)/Bi_2Te_3 (10 QL) heterostructures. **(c)** The fitting results of the EDC for heterostructure $x = 2$ nm. The black line is the EDC, the orange lines represent the muti-Lorentzian fitting of the individual peaks, and the red line is the total fitting line of the MDC. The green arrow indicates the peak position of the band, corresponding to the yellow dot in Figure 3(a).

S11. ARPES results of GeTe (x nm)/Bi_2Te_3 (1 QL) heterostructures

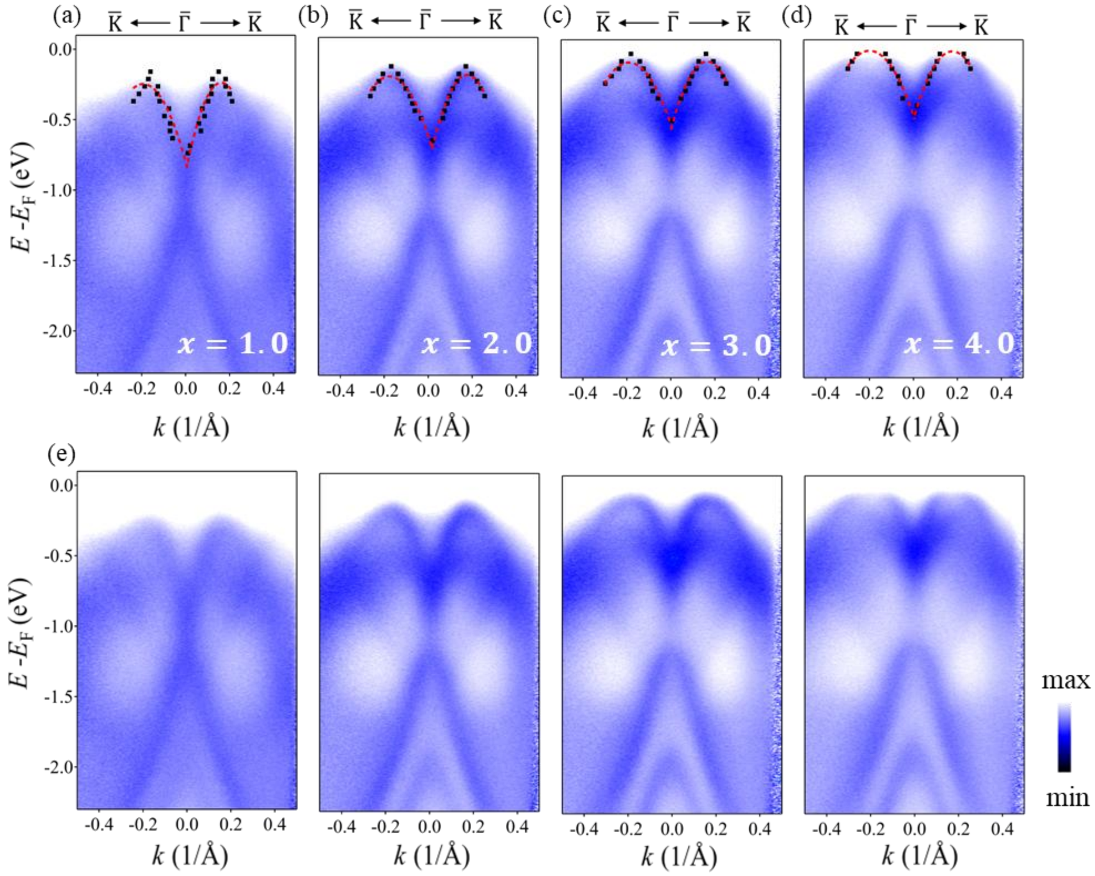

Figure S11 (a-d) ARPES spectra of GeTe (x nm)/Bi_2Te_3 (1 QL) heterostructures with GeTe thickness x=1.0, 2.0, 3.0, and 4.0 nm, respectively. The black dots represent the peak positions of the momentum distribution curves (MDCs). The red dashed lines are the fitted lines for Rashba bands. **(e)** The raw ARPES spectra of Figure S11 (a-d).

References

[1] P. A. Vermeulen, J. Mulder, J. Momand, B. J. Kooi, *Nanoscale* **2018**, 10, 1474.

[2] M. Kriener, M. Sakano, M. Kamitani, M. S. Bahramy, R. Yukawa, K. Horiba, H. Kumigashira, K. Ishizaka, Y. Tokura, Y. Taguchi, *Phys Rev Lett* **2020**, 124, 047002.

[3] Y. Li, Y. Li, P. Li, B. Fang, X. Yang, Y. Wen, D. X. Zheng, C. H. Zhang, X. He, A. Manchon, Z. H. Cheng, X. X. Zhang, *Nat. Commun.* **2021**, 12, 540.

[4] R. Yoshimi, K. Yasuda, A. Tsukazaki, K. S. Takahashi, M. Kawasaki, Y. Tokura, *Sci. Adv.* **2018**, 4, eaat9989.

[5] B. Croes, A. Llopez, C. Tagne-Kaegom, B. Tegomo-Chiogo, B. Kierren, P. Müller, S. Curiotto, P. Le Fèvre, F. Bertran, A. Saúl, Y. Fagot-Revurat, F. Leroy, F. Cheynis, *Nano Lett.* **2024**, 24, 13224.

[6] K. Premasiri, S. K. Radha, S. Sucharitakul, U. R. Kumar, R. Sankar, F. C. Chou, Y. T. Chen, X. P. A. Gao, *Nano Lett.* **2018**, 18, 4403.

[7] S. Chatterjee, S. Khalid, H. S. Inbar, A. Goswami, T. Guo, Y.-H. Chang, E. Young, A. V. Fedorov, D. Read, A. Janotti, C. J. Palmstrøm, *Sci. Adv.* **2021**, 7, eabe8971.

[8] V. L. Deringer, M. Lumeij, R. Dronskowski, *The Journal of Physical Chemistry C* **2012**, 116, 15801.

[9] M. Liebmann, C. Rinaldi, D. Di Sante, J. Kellner, C. Pauly, R. N. Wang, J. E. Boschker, A. Giussani, S. Bertoli, M. Cantoni, L. Baldrati, M. Asa, I. Vobornik, G. Panaccione, D. Marchenko, J. Sanchez-Barriga, O. Rader, R. Calarco, S. Picozzi, R. Bertacco, M. Morgenstern, *Adv. Mater.* **2016**, 28, 560.

[10] J. Krempaský, H. Volfová, S. Muff, N. Pilet, G. Landolt, M. Radović, M. Shi, D. Kriegner, V. Holý, J. Braun, H. Ebert, F. Bisti, V. A. Rogalev, V. N. Strocov, G. Springholz, J. Minár, J. H. Dil, *Phys. Rev. B* **2016**, 94, 205111.

[11] Y. Y. Li, G. Wang, X. G. Zhu, M. H. Liu, C. Ye, X. Chen, Y. Y. Wang, K. He, L. L. Wang, X. C. Ma, H. J. Zhang, X. Dai, Z. Fang, X. C. Xie, Y. Liu, X. L. Qi, J. F. Jia, S. C. Zhang, Q. K. Xue, *Adv. Mater.* **2010**, 22, 4002.

309
